# Environmental and molecular control of tissue-specific ionocyte differentiation in zebrafish

**DOI:** 10.1242/dev.202809

**Published:** 2024-10-22

**Authors:** Julia Peloggia, Mark E. Lush, Ya-Yin Tsai, Christopher Wood, Tatjana Piotrowski

**Affiliations:** Stowers Institute for Medical Research, Kansas City, MO 64110, USA

**Keywords:** Plasticity, Ion homeostasis, Ionocytes, Lateral line, Zebrafish, Notch pathway

## Abstract

Organisms cope with environmental fluctuations and maintain fitness in part via reversible phenotypic changes (acclimation). Aquatic animals are subject to dramatic seasonal fluctuations in water salinity, which affect osmolarity of their cells and consequently cellular function. Mechanosensory lateral line hair cells detect water motion for swimming behavior and are especially susceptible to salinity changes due to their direct contact with the environment. To maintain hair cell function when salinity decreases, neuromast (Nm)-associated ionocytes differentiate and invade lateral line neuromasts. The signals that trigger the adaptive differentiation of Nm ionocytes are unknown. We demonstrate that new Nm ionocytes are rapidly specified and selectively triggered to proliferate by low Ca^2+^ and Na^+^/Cl^−^ levels. We further show that Nm ionocyte recruitment and induction is affected by hair cell activity. Once specified, Nm ionocyte differentiation and survival are associated with sequential activation of different Notch pathway components, a process different from other tissue-specific ionocytes. In summary, we show how environmental changes activate a signaling cascade that leads to physiological adaptation. This may prove essential for survival not only in seasonal changing environments but also in changing climates.

## Teaser

**Summary:** Ionocyte progenitors invade lateral line sensory organs in response to low medium salinity or pH, which are environmental changes that trigger the Notch signaling pathway and orchestrate this physiological adaptation.

## INTRODUCTION

Organisms have evolved various strategies to thrive in fast-changing environments. One crucial mechanism that enhances their adaptability is phenotypic plasticity, where the same genotype leads to different phenotypes in an environment-dependent manner (Beaman et al., 2016; Kelly et al., 2012). Many examples of phenotypic plasticity have been studied in detail, such as the adaptation of organisms to different temperatures, body size regulation in sea urchins, fur coat changes of arctic foxes, hypoxia tolerance in fishes and responses of organisms to osmotic stresses (Agrawal, 2001; Guh et al., 2015; Hwang, 2009; Hwang and Chou, 2013; Kelly et al., 2012).

In mammals and other terrestrial vertebrates, osmoregulation, which is the control of water and ion concentrations in the body, occurs mainly through dietary intake and urine production in the kidney (Schmidt-Nielsen, 1997). Although this is a systemic regulation, multiple tissues have additional mechanisms to fine-tune the control of the ionic balance and osmoregulation. Their ion composition is maintained in part by tissue-specific ion-regulating cells named ionocytes. Ionocytes express ion channels that actively transport ions across the cell membranes and establish proper ionic concentration and osmotic pressures (Hwang and Chou, 2013; Shah et al., 2022). In mammals, locally acting tissue-specific ion-regulating cells have been identified in the ear, epididymis, kidney and lungs, and loss of function of ion channels highly expressed in these cells leads to hearing loss, infertility, renal tubular acidosis and cystic fibrosis, respectively (Honda et al., 2017; Kriz and Kaissling, 2008; Montoro et al., 2018; Pou Casellas et al., 2023; Rao et al., 2019).

Aquatic animals, particularly those living in freshwater habitats, are subjected to much greater fluctuations in environmental salinity and pH (Kültz, 2015). Consequently, these organisms require fast and robust adaptation mechanisms to thrive in these dynamic conditions. To control ion homeostasis and osmoregulation, teleost fishes also rely on ionocytes (Guh et al., 2015; Hwang and Chou, 2013). To date, five main ionocyte subtypes have been identified in zebrafish and are classified based on their expression of different channels and their specific roles in regulating ions: HR (H^+^ secretion/Na^+^ uptake/NH4^+^ excretion), NaR (Ca^2+^ uptake), NCC (Na^+^/Cl^−^ uptake), KS (K^+^ secretion) and SLC26 ionocytes (Cl^−^ uptake/ HCO3^−^ secretion) (Yan and Hwang, 2019). Although ionocytes were first described in the eel gill epithelium (Keys and Willmer, 1932), it is now appreciated that they also associate with organs such as the embryonic skin of fish and frogs, and the zebrafish sensory lateral line (Farquhar and Palade, 1965; Peloggia et al., 2021).

**Fig. 1.**
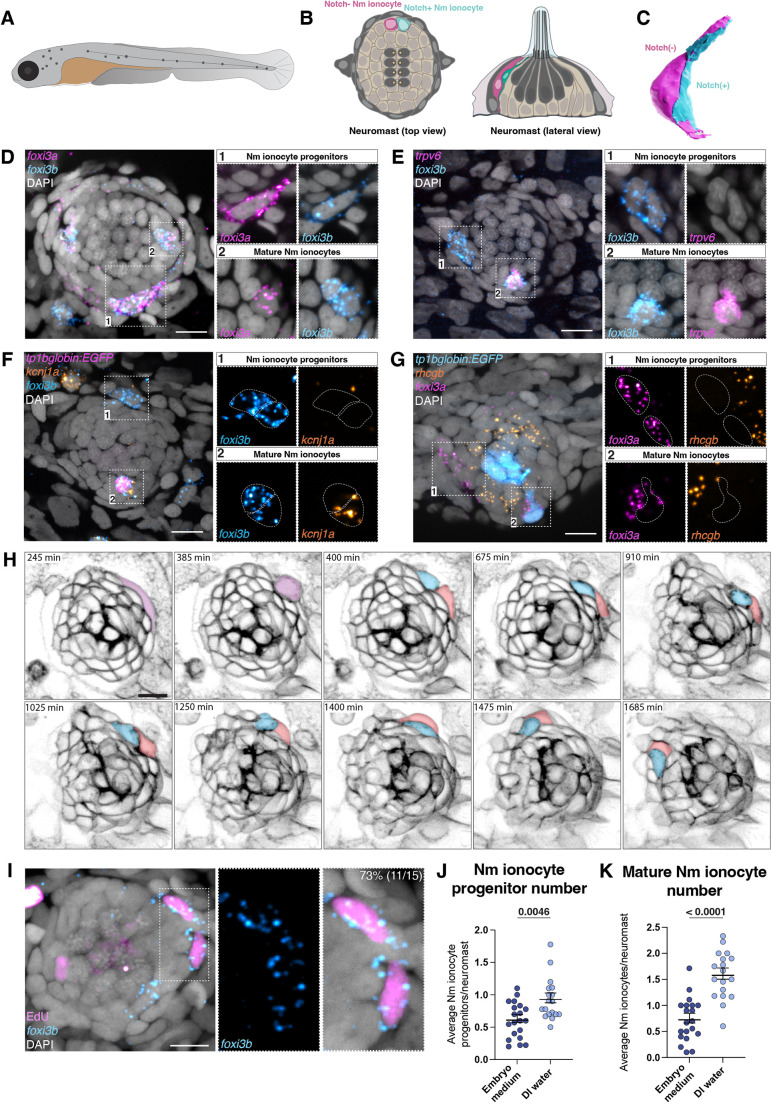
**Nm ionocyte progenitors are *foxi3a/foxi3b*-positive cells derived from one cell division event.** (A) Schematic of a zebrafish larva at 5 dpf. Lateral line neuromasts are labeled in dark grey*.* (B) Schematic of a neuromast top view (left) and lateral view (right). Nm ionocyte pair is labeled in magenta and cyan. (C) 3D modeling of Nm ionocyte pair. (D) Hybridization chain reaction (HCR) labeling of transcription factors *foxi3a* and *foxi3b* shows two mature Nm ionocytes inside a lateral line neuromast (2) and a ventrally located double-positive progenitor adjacent to the neuromast (1). (E) Maximum intensity projection of HCR for *foxi3b* and *trpv6* labeling a pair of progenitors and one of mature Nm ionocytes. (F) Maximum intensity projection of HCR for *foxi3a* and *kcnj1a* in the Notch reporter (*tp1bglobin:EGFP*) background. (G) Maximum intensity projection of *foxi3a* and *rhcgb* HCRs in the Notch reporter (*tp1bglobin:EGFP*) background. (H) Maximum intensity projection of still images of a time-lapse of *cldnb:lyn-EGFP* fish at 3 dpf. A pair of cells invades a neuromast. (I) EdU incorporation in combination with *foxi3b* HCR (J) Average number of Nm ionocyte progenitors per neuromast for embryos incubated in embryo medium (*n*=20 larvae) or in DI water for 48 h (*n*=18 larvae; Mann–Whitney test). (K) Average number of mature Nm ionocyte per neuromast for embryos incubated in embryo medium or in DI water for 48 h (unpaired *t*-test). Data are mean±s.e.m. Scale bars: 10 µm.

The zebrafish lateral line is a mechanosensory organ composed of multiple sensory units called neuromasts distributed along the trunk and head (Fig. 1A,B). These neuromasts contain sensory hair cells that detect water flow and help with orientation, prey detection and predator avoidance (Lush and Piotrowski, 2014). We have previously demonstrated that upon changes in salinity and pH, basal skin cells migrate, invade mature lateral line neuromasts and differentiate into neuromast-associated ionocytes (Nm ionocytes, Fig. 1B,C) (Peloggia et al., 2021). We named this process ‘adaptive cell invasion’ and showed that Nm ionocytes help to fine tune the activity of hair cells, ensuring their functionality across a wide range of environmental conditions. However, how environmental changes are detected and what signals are required to specify Nm ionocytes are still not understood.

Here, we identify for the first time the progenitors that give rise to mature Nm ionocytes and show that their survival is dependent on Notch signaling. We demonstrate that Nm ionocyte specification is triggered by absolute levels of salinity and that the salinity sensor discriminates between different ions. Finally, we show that hair cell function plays a role in the control of Nm ionocyte number. Collectively, we elucidate the earliest cellular and molecular events required for Nm ionocyte specification and differentiation, and shed light on the mechanisms required for sensory organ adaptation to different environmental changes.

## RESULTS

### Identification of Nm ionocyte progenitors

We previously showed that decreased salinity and pH lead to the adaptive differentiation of basal stem cells into a pair of Nm ionocytes, which invade lateral line neuromasts (Peloggia et al., 2021). How zebrafish detect environmental changes and activate this adaptive response is still unknown. Here, we sought to identify the early steps of ionocyte induction and differentiation. The transcription factors *foxi3a* and *foxi3b* (*foxi3a/3b*) are major regulators of zebrafish skin and Nm ionocyte development, and their loss inhibits ionocyte differentiation (Hsiao et al., 2007; Jänicke et al., 2007; Peloggia et al., 2021). To test whether *foxi3a/3b* mRNAs label Nm ionocyte progenitors before invasion, we performed whole-mount hybridization chain reaction (HCR) RNA fluorescent *in situ* hybridization. We observed cells immediately adjacent to neuromasts that co-express *foxi3a/3b* (Fig. 1D, inset 1). Inside the neuromast, *foxi3a* is expressed in only one Nm ionocyte (HR-like and Notch negative), whereas *foxi3b* is expressed in both cells of the mature Nm ionocyte pair (Fig. 1D, inset 2)*.* This is consistent with the molecular similarities of these cells with HR and NaR skin ionocytes (Fig. S1A,B) (Peloggia et al., 2021). These data indicate that *foxi3a/foxi3b*-expressing cells adjacent to neuromasts could be the Nm ionocyte progenitors.

To further investigate the identity and differentiation state of the cells while outside neuromasts, we performed HCRs for genes expressed in differentiated ionocytes, in combination with a Notch reporter (*tp1bglobin:EGFP*) that labels one cell of the mature Nm ionocyte pair. The Ca^2+^ channel *trpv6* is expressed both in skin and mature Nm ionocytes but not in the putative progenitors outside the neuromasts (Fig. 1E and Fig. S1C). Likewise, the ion channel genes *kcnj1a.1*, *rhcgb* and *slc4a1b* are only expressed in differentiated ionocytes (Fig. 1F,G and Fig. S1D-F). These findings suggest that the cells outside the neuromasts are not fully differentiated or functional, and could be the progenitors that give rise to Nm ionocytes. Additionally, these cells are crescent shaped and morphologically distinct from mature skin ionocytes, which are bigger, with round nuclei and square cell morphology (Fig. S1C-H). These morphological differences further support the notion that these cells are not a subtype of differentiated skin ionocytes, but Nm ionocyte progenitors. To confirm that Nm ionocytes are derived from undifferentiated basal skin cells and not mature skin ionocytes, we performed time-lapse analysis of labeled mature ionocytes using a short pulse of MitoTracker (Esaki et al., 2009; Shir-Mohammadi and Perry, 2020). We observed that mature skin ionocytes are not motile and do not invade lateral line neuromasts (Fig. S1I, Movie 1). Instead, new Nm ionocytes derive from pairs of smaller unlabeled cells (Fig. S1J).

The fact that mature Nm ionocytes are always present in pairs implies they might arise by cell division of a single progenitor or stem cell. To test whether Nm ionocyte progenitors divide, we performed an ethynyl-2′-deoxyuridine (EdU) incorporation assay followed by *foxi3b* HCR. We observed that the majority of Nm ionocyte progenitors were EdU positive, and often appeared in pairs (Fig. 1I). Time-lapse analyses of Nm ionocyte development also showed that a single precursor divides and that the resulting pair invades neuromasts (Fig. 1H and Movie 2), confirming that the pair of mature Nm ionocytes arises by cell division of a single progenitor cell. Taken together, these data show that Nm ionocyte progenitors are pairs of *foxi3a/foxi3b*-positive cells that derive from a single cell division event.

### Nm ionocyte progenitors are induced adjacent to neuromasts by low salinity

Nm ionocyte differentiation is an adaptive behavior triggered by changes in media salinity and pH (Peloggia et al., 2021). We next tested whether the number of *foxi3a/foxi3b-*positive Nm ionocyte progenitors is dependent on salinity. Incubation of zebrafish in deionized water (DI water) for 48 h increases the total progenitor number per neuromast (average number), as well as the number of neuromasts with Nm ionocyte progenitors (frequency) (Fig. 1J and Fig. S1K). Consistent with the higher progenitor number, the average number and frequency of mature Nm ionocytes also increase after 48 h of a decrease in salinity (Fig. 1K and Fig. S1L), similar to our earlier findings (Peloggia et al., 2021). The increase in Nm ionocyte progenitor frequency in low salinity suggests that new progenitors are induced in response to environmental stimuli. Additional analyses of *foxi3a* and *foxi3b* HCRs show the presence of a subset of cells surrounding the neuromasts that express only *foxi3a*, whereas few *foxi3b*-only cells were detected (Fig. S1M,N). This suggests that, as in skin ionocytes, *foxi3a* is activated before *foxi3b* (Hsiao et al., 2007; Jänicke et al., 2007; Peloggia et al., 2021). Moreover, we detected individual *foxi3a*- and *foxi3a/b*-positive cells, which indicates these transcription factors are upregulated before cell division (Fig. 1D and Fig. S1M). We conclude from these data that new Nm ionocyte progenitors are induced adjacent to neuromasts by low salinity, and that *foxi3a* and *foxi3b* are upregulated before cell division.

### Components of the Notch signaling pathway are expressed in a precise spatio-temporal pattern during Nm ionocyte differentiation

Once inside neuromasts, each cell of the progenitor pair adopts a specific ionocyte fate, shown by the differential expression of transcription factors and ion channels (Peloggia et al., 2021). To address the molecular basis of Nm ionocyte fate determination, we focused on the Notch signaling pathway, as its expression has been observed in Nm ionocytes. Notch signaling is an ancestral pathway that controls cell identity and cell fate decisions via the interaction of a ligand with a receptor expressed in a neighboring cell (Gazave et al., 2009). Their interaction results in the cleavage of the Notch receptor intracellular domain (NICD), which translocates to the nucleus and drives changes in gene expression (Henrique and Schweisguth, 2019; Sjöqvist and Andersson, 2019). Zebrafish possess four receptors and nine different Notch ligands (five Delta and four Jagged ligands), and interactions of Notch receptors with different ligands lead to distinct downstream outputs (Nandagopal et al., 2018).

**Fig. 2.**
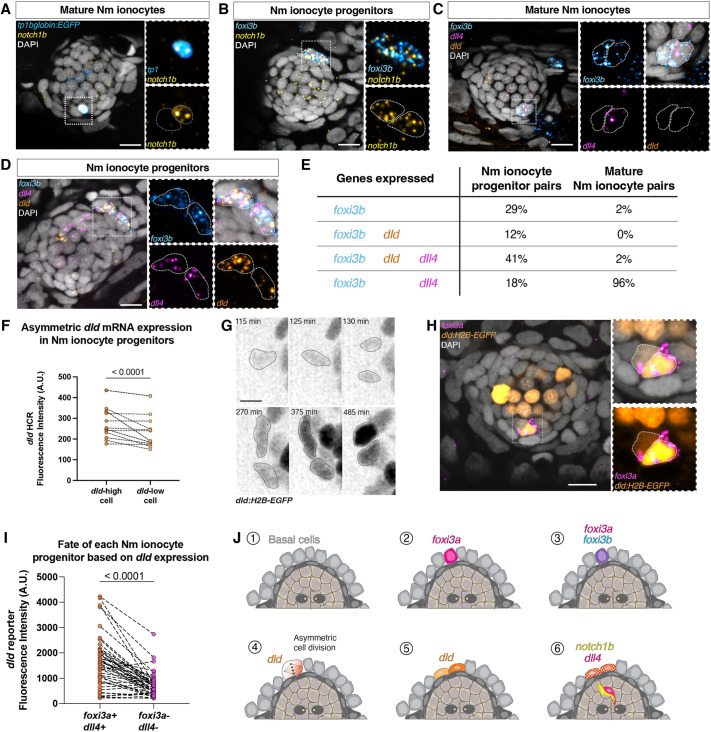
**A switch of Notch ligand expression is associated with ionocyte differentiation and invasion of lateral line organs in zebrafish.** (A) Confocal image of *notch1b* hybridization chain reactions (HCRs) in mature Nm ionocytes and (B) Nm ionocyte progenitors. (C,D) Representative images of triple HCR for *foxi3b*, *dll4* and *dld* expression in (C) mature Nm ionocytes and (D) Nm ionocyte progenitors. (E) Percentages of Nm ionocyte pairs with expression of genes *foxi3b*, *dll4* and *dld* (*n*=51 mature Nm ionocytes and 17 progenitor pairs). (F) Quantification of *dld* mRNA expression between the two cells of the progenitor pair (unpaired *t*-test). (G) Maximum intensity projection of *Tg(dld:H2B-EGFP)^psi84Tg^* combined with HCR for *foxi3a*. The *dld* reporter labels both Nm ionocytes and lateral line hair cells. (H) Time-lapse analysis of *dld:H2B-EGFP* shows that *dld* is upregulated before cell division. Gamma was changed in the *dld:H2B-EGFP* channel to allow the visualization of dim structures. (I) Quantification of EGFP fluorescence in each Nm ionocyte of the pair and its relation to *foxi3a* expression (*n*=45 pairs of cells, 24 larvae; Wilcoxon matched-pairs signed rank test). (J) Summary of gene expression during Nm ionocyte development. Scale bars: 10 µm in A-D,H; 5 µm in G. All images are from 5 dpf larvae.

One of the two mature Nm ionocytes expresses the receptor *notch1b* (Fig. 2A) (Peloggia et al., 2021). To test whether other Notch receptors are present in mature Nm ionocytes and progenitors, we performed HCRs of *notch1a*, *notch1b* and *notch3* in combination with a Notch reporter (*tp1bglobin:EGFP*) or *foxi3b* HCR, respectively. *notch1b* is expressed in both *foxi3b*-positive progenitors (Fig. 2B), whereas *notch1a* and *notch3* are expressed in neither progenitors nor mature Nm ionocytes (Fig. S2A-E). This contrasts with the skin, where *notch1a* and *notch3*, but not *notch1b*, play a role in ionocyte specification (Hsiao et al., 2007; Jänicke et al., 2007). To determine which ligands are expressed in mature Nm ionocytes, we performed HCR for all known Delta and Jagged ligands. We detected expression of only *dll4* in the HR-like Nm ionocyte, and no other ligands in the mature pair (Fig. 2C and Fig. S2F). Thus, mature Nm ionocyte pairs express a *notch1b*-*dll4* receptor-ligand combination. To determine whether Notch signaling could be involved in specifying distinct fates in the progenitors, we analyzed expression of ligands by HCR before invasion of neuromasts. We observed expression of *dll4* in both progenitor cells, as well as of a second ligand, *dld* (Fig. 2D and Fig. S2G). However, not all *foxi3b*-positive progenitors expressed these two Notch ligands. To determine the dynamics of *dll4* and *dld* expression in both progenitors, we analyzed their frequency in combination with *foxi3b*. We also observed progenitors that express only *foxi3b*, suggesting it is transcribed before Notch ligands are expressed (Fig. 2E). A small percentage of progenitors co-express either *foxi3b/dld* or *foxi3b/dll4*, and almost half of the progenitors express all three genes (*foxi3b*, *dld* and *dll4*). The most parsimonious interpretation is that *dld* is expressed before *dll4* and is downregulated before neuromast invasion.

We noticed an asymmetry in the levels of *dld* expression in progenitors shown by HCR (Fig. 2D and F). To investigate the dynamic expression of *dld* in more detail, we generated a reporter line by knocking-in H2B-EGFP into the 5′UTR of *dld* (*dld:H2B-EGFP*) with CRISPR-Cas12a (Fig. S4A-C)*.* Time-lapse analyses of the *dld:H2B-EGFP* reporter show that *dld* is expressed before mitosis and that the expression of GFP becomes asymmetric after cell division (Fig. 2G, Movie 3), resulting in a *dld:H2B-EGFP*^high^ and a *dld:H2B-EGFP*^low^ cell. The histone-tagged GFP is stable, making Nm ionocytes fluorescent as they invade neuromasts and differentiate, and the GFP can therefore be used as a lineage tracer (Fig. S4A,B). HCR for *foxi3a* in *dld:H2B-EGFP* transgenic larvae shows that the *dld:H2B-EGFP*^high^ cell differentiates into the *foxi3a*-positive Nm ionocyte, which also expresses *dll4* (Fig. 2H,I and Fig. S2F).

Altogether, our results demonstrate that different Notch pathway components are dynamically expressed during Nm ionocyte differentiation. *foxi3a* and *foxi3b* are expressed in the progenitor cells before cell division and before Notch signaling. *dld* is then transiently expressed in both daughter cells as they divide and is downregulated as the progenitors invade neuromasts and differentiate. Concomitant with the *dld* pulse, *dll4* and *notch1b* are upregulated in both progenitors but each becomes restricted to one of the mature cells, with *dll4* being restricted to the *dld*^high^ cell and *notch1b* to the *dld*^low^ cell (Fig. 2J).

Notch signaling culminates in the NICD-dependent transcriptional activation of several genes, including genes in the *hairy/enhancer of split* (*her*) family (Jarriault et al., 1995). These transcription factors repress genes in the Notch-positive cell that are activated in the Delta/Jagged-positive cell. We identified candidate downstream factors in our previously published single cell RNA sequencing data (scRNAseq) of ionocytes (Fig. S3A) and performed HCRs. *hes6* and *her15* are expressed in mature NaR-like Nm ionocytes, and *her15* is expressed in some, but not all, progenitors, while the remaining candidates are not detected in Nm ionocytes (Fig. S3B-H). Additionally, *her15* expression is Nm ionocyte specific, as it is not detected in skin ionocytes (Fig. S3I,J). These results show that Notch signaling activates different downstream targets in the neuromast and skin ionocyte populations, and that *her15* is a specific target of *notch1b* and/or *dll4* signaling in Nm ionocytes.

### *dll4* regulates ionocyte survival

**Fig. 3.**
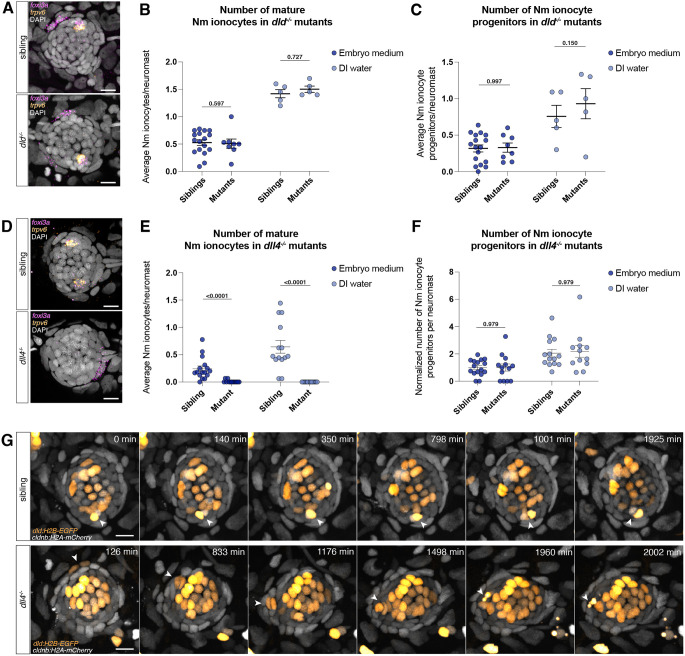
***dll4* affects Nm ionocyte differentiation but not progenitor specification.** (A) Confocal images of neuromasts of wild-type siblings (top) and *dld* mutants (bottom) showing the presence of mature Nm ionocytes and progenitors. (B,C) Number of (B) mature Nm ionocytes and (C) progenitors in both siblings (embryo medium, *n*=17 larvae; DI water, *n*=5 larvae) and *dld* mutants in embryo medium (*n*=8 larvae; DI water, *n*=5 larvae; unpaired *t*-test) raised in either embryo medium or incubated in DI water. (D) Confocal images of neuromasts of wild-type siblings (top) and *dll4* mutants (bottom) showing the presence of mature Nm ionocytes and a progenitor, respectively. (E,F) Number of (E) mature Nm ionocytes and (F) progenitors in both siblings (embryo medium, *n*=16 larvae; DI water, *n*=14 larvae) and *dll4* mutants (embryo medium, *n*=13 larvae; DI water, *n*=12 larvae; Mann–Whitney test) raised in different salinities, normalized to embryo medium control. (G) Time-lapse still images showing a mature Nm ionocyte in 4 dpf siblings and an invading Nm ionocyte pair in *dll4* mutants that undergoes cell death. Cells are labeled with *dld:H2B-EGFP* and *cldnb:H2A-mCherry*. Scale bars: 10 µm. Data are mean±s.e.m. Data in A-F are from 5 dpf larvae.

To dissect the roles of the Notch ligands *dll4* and *dld* in Nm ionocyte specification and differentiation, we generated *dld* and *dll4* mutants using CRISPR/Cas12a (Figs S4D-F and S5A-C). Even though the *dld* mutant shows somitogenesis defects similar to other alleles (Van Eeden et al., 1996) (Fig. S4E), the number and type of ionocytes do not change in the skin or neuromasts (Fig. 3A-C and Fig. S4G-I). We conclude that *dld* is not essential for Nm ionocyte determination and differentiation. In contrast, *dll4* mutants show a drastic reduction of Nm ionocytes both in embryo medium and DI water compared with wild-type siblings (Fig. 3D,E). However, the number of Nm ionocyte progenitors does not change in *dll4* mutants (Fig. 3F). Thus, *dll4* is required for maturation of Nm ionocytes, but not for progenitor specification.

To determine why *dll4* mutants lack mature Nm ionocytes, we performed time lapse analyses of mutants and siblings using the *dld:H2B-EGFP* reporter that labels Nm ionocytes and hair cells. We observed Nm ionocytes invading neuromasts in both siblings and mutants from 3 to 5 dpf. However, we observed a higher incidence of Nm ionocyte death in mutants compared with wild-type larvae, indicating that *dll4* plays a role in cell survival following invasion (Fig. 3G, Movies 4 and 5). This is consistent with our previous results that found that pharmacological inhibition of Notch signaling leads to death of mature ionocytes (Peloggia et al., 2021). It is also possible, however, that the cell death phenotype we observe in *dll4* mutants is a consequence of aberrant differentiation. The lack of mature Nm ionocytes in *dll4* mutants prevents us from assessing the role of *dll4*-*notch1b* signaling in Nm ionocyte fate specification.

Previous work described that *dll4* is not expressed in the skin and it is not required for skin ionocyte differentiation (Hsiao et al., 2007; Jänicke et al., 2007). However, we observed that although the total skin ionocyte (HR and NaR) density was not changed in 5 dpf *dll4* mutants (Fig. S5D), the proportion of ionocyte subtypes was altered. *foxi3a*-positive HR ionocytes increase in density in the mutants, whereas *trpv6*-positive NaR skin ionocytes are totally absent in the skin of *dll4* mutants and are present only in the gills (Fig. S5E,F). Indeed, a subset of skin ionocytes express *dll4* at 5 dpf (Fig. S5G). Taken together, our results show that *dll4* regulates the proportion of HR and NaR skin ionocytes.

### New Nm ionocyte progenitors are rapidly induced by low salinity

**Fig. 4.**
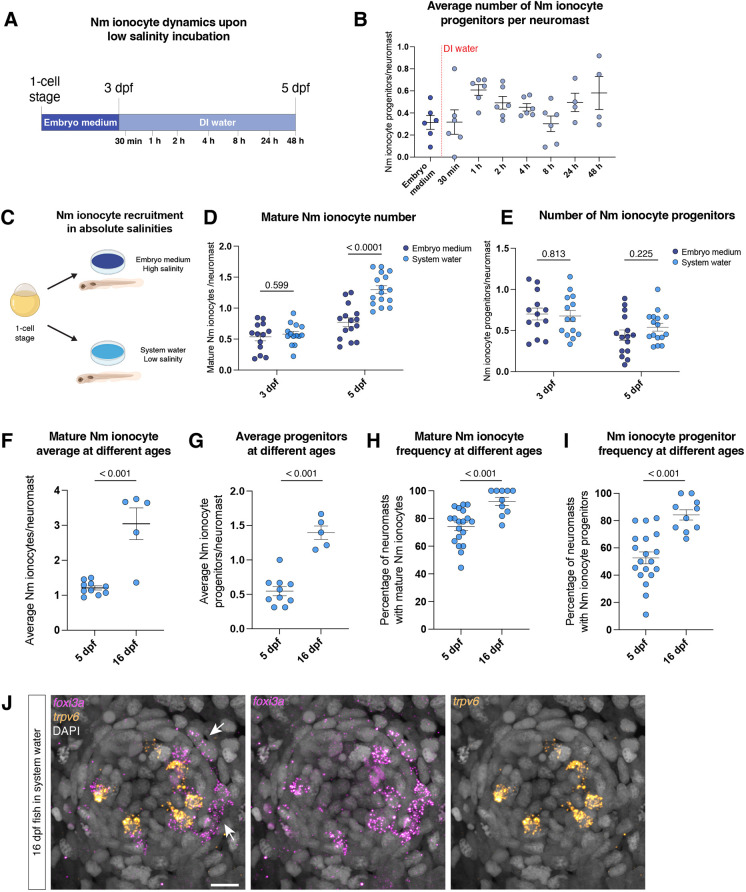
**Nm ionocytes are rapidly induced by absolute low salinity.** (A) Schematic representation of the time course experiment. (B) Average number of Nm ionocyte progenitors per neuromast after salinity decrease. (C) Schematic representation of absolute salinity experiment. (D,E) Average number of mature ionocytes (D) and progenitors per neuromast (E) of fish raised in different salinities at 3 and 5 dpf (unpaired *t*-test). (F,G) Average number of mature ionocytes (F) and progenitor number (G) in 5 dpf (*n*=10 larvae) and 16 dpf (*n*=5 larvae) larvae raised in system water (unpaired *t*-test). (H,I) Frequency of mature (H) and Nm ionocyte progenitors (I) at 5 and 16 dpf raised in system water (unpaired *t*-test). (J) Representative hybridization chain reaction showing mature and Nm ionocyte progenitors in a neuromast of a 16 dpf fish. Arrows indicate Nm ionocyte progenitors. Scale bar: 10 µm. Data are mean±s.e.m.

It is not known how environmental stimuli are sensed at a cellular and molecular level, and translated into progenitor activation and ionocyte differentiation. To investigate this process, we first characterized Nm ionocyte differentiation dynamics upon salinity decrease, specifically the upregulation of *foxi3a/b* transcription factors. Time course analyses after DI water incubation showed that *foxi3a/b*-positive Nm ionocyte progenitors were quickly induced after salinity decrease, with a peak around 1 h post incubation (Fig. 4A,B). This rapid response suggests that the link between the sensor and the effector likely does not rely on detection by sensory systems, processing in higher brain centers and relay through hormonal changes, but rather on more direct components. Additionally, we observed that the number of progenitors oscillates over time, with a peak after DI water incubation and subsequent decrease around 4-8 h after incubation, which likely correlates with these cells differentiating and invading the neuromasts. Progenitor oscillation is also observed in time lapses, where new Nm ionocyte progenitors upregulate *dld:H2B-EGFP* and invade neuromasts, but new progenitors are not detected for several hours based on *dld:H2B-EGFP* expression (Movie 3).

A fast upregulation of Nm ionocyte progenitors could be caused by a stress response after salinity changes. To test whether incubation in low salinity triggers stress response pathways, we tested: (1) the activation of glucocorticoid (GR) signaling based on the expression of the downstream factor *dusp1* (Abraham et al., 2006; Xavier et al., 2016); and (2) expression of components of the immediate early response AP-1 complex – *fosab* and *junba*. GR induces skin ionocyte proliferation upon salinity changes (Cruz et al., 2013; Guh and Hwang, 2017), and AP-1 is activated in neuromasts upon hair cell death (Baek et al., 2022). DI water incubation does not induce *dusp1* transcription in the skin or in the neuromast (Fig. S6A-C). We observed an increase in *fosab* mRNA expression 15 and 30 min after DI water incubation, and a slight transient increase of *junba* after 15 min incubation in DI water (Fig. S6D-F). We next checked the expression of these genes in embryos incubated in system water. System water has an intermediate salinity compared with embryo medium and DI water, and, consequently, the resulting frequency of Nm ionocytes. However, incubation in system water does not lead to expression of *fosab* and *junba* (Fig. S6D-F). This suggests that, although components of the AP-1 pathway are transcribed after DI water incubation, they may not be required for activating Nm ionocyte progenitors.

We next sought to test whether Nm ionocyte induction requires salinity changes or whether it is triggered by absolute levels of salinity. We incubated zebrafish embryos in different media starting at the one-cell stage and quantified Nm ionocyte numbers at 3 and 5 dpf (Fig. 4C). At 3 dpf, numbers of mature Nm ionocytes and progenitors are not different between larvae raised in embryo medium (high salinity) or zebrafish system water (low salinity). However, at 5 dpf, mature Nm ionocyte numbers in larvae raised in low salinity increase (Fig. 4D), while progenitor Nm ionocyte numbers does not change (Fig. 4E). Furthermore, we do not see the activation of stress response pathways in constant low salinity (Fig. S6G-I). These data demonstrate that the sensor that controls Nm ionocyte numbers detects absolute levels of salinity and not a change in salinity.

Mature Nm ionocytes increase in number from 3 to 5 dpf, both in embryo medium and system water. The number of progenitors, however, does not change within this time frame. This could be due to the progenitor turnover mentioned above. We have previously demonstrated that the number of mature Nm ionocytes increases in adult animals (Peloggia et al., 2021). To assess whether progenitor numbers also change with age, we performed HCR for *foxi3a* and *trpv6* in 16 dpf juveniles. We observed that average mature Nm ionocyte number is higher in juveniles than in larvae, but the number of progenitors does not scale to the same proportions, with most neuromasts still having one or two pairs of progenitors but containing up to six mature Nm ionocyte pairs (Fig. 4F,G). Meanwhile, the frequency of mature Nm ionocytes and progenitors is higher in juveniles when compared with 5 dpf larvae in the same salinity (Fig. 4H-J). We conclude from these data that progenitors are likely a finite population around neuromasts and that the increase in adult mature Nm ionocyte numbers is due to increased invasion events.

### Differentiation of new Nm ionocytes is selectively triggered by different ions

**Fig. 5.**
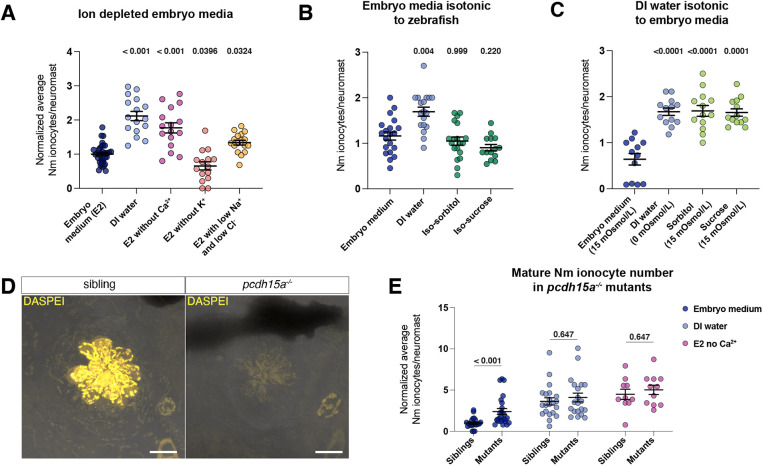
**Nm ionocytes are triggered by low levels of Ca^2+^ and Na^+^/Cl^−^ ions.** (A) Number of mature Nm ionocytes in regular embryo medium, DI water and embryo medium depleted of either calcium or potassium, or with low levels of sodium and chloride (embryo medium, *n*=34 larvae; DI water, *n*=16 larvae; no Ca^2+^, *n*=16 larvae; no K^+^, *n*=15 larvae; low Na^+^/Cl^−^, *n*=17 larvae; one-way ANOVA, normalized to embryo medium control). (B) Number of mature Nm ionocytes in isotonic solutions made with sorbitol or sucrose (embryo medium, *n*=20 larvae; DI water, *n*=18 larvae; iso-sorbitol, *n*=19 larvae; iso-sucrose, *n*=14 larvae; one-way ANOVA and Dunnett's multiple comparisons test). (C) Number of mature Nm ionocytes in DI water supplemented with sorbitol or sucrose to the osmolarity of embryo medium (embryo medium, *n*=12 larvae; DI water, *n*=13 larvae; sorbitol, *n*=13 larvae; sucrose, *n*=13 larvae; one-way ANOVA and Dunnett's multiple comparisons test). (D) Active hair cells are labeled using DASPEI in *pcdh15a* siblings, but not in mutants. Scale bars: 10 µm. (E) Number of mature Nm ionocytes in siblings and *pcdh15a* mutants in embryo medium (*n*=22 siblings, 24 mutants), DI water (*n*=21 siblings, 20 mutants) and no Ca^2+^ (*n*=10 siblings, 12 mutants) (multiple Mann–Whitney test, normalized to embryo medium control). Data are mean±s.e.m.

Thus far, we determined that the salinity sensor responds fast, does not require a stress response and detects absolute salinity. We next asked which specific ions are being detected, and incubated embryos for 48 h in embryo media lacking individual ions. Incubation in medium lacking Ca^2+^ ions leads to an increase of mature Nm- and skin NaR ionocytes (Fig. 5A and Fig. S7A). Additionally, lack of the majority of Na^+^ and Cl^−^ ions in the medium also leads to an increase in Nm ionocyte number, but to a lesser extent, even though Na^+^ and Cl^−^ ions are present in much higher concentrations in regular medium than Ca^2+^. Meanwhile, K^+^ depletion does not lead to an increase of Nm ionocytes. These results demonstrate that specific ions, and not general changes in medium ionic concentration, play a role in inducing new Nm ionocytes.

Zebrafish, like other freshwater fish, live in a hypotonic environment and must balance the undesired uptake of water. Embryo medium (15 mOsmol/l) is hypotonic compared with zebrafish interstitial fluid (∼270 mOsmol/l), which causes undesired water influx that is compensated for by the kidney (Potts, 1984). To address whether water influx is required for Nm ionocyte induction and differentiation, we incubated larvae in isotonic embryo medium supplemented with either D-sorbitol or sucrose to achieve interstitial fluid osmolarity (Gault et al., 2014; Kennard and Theriot, 2020). Larvae raised in isotonic solution show similar numbers of Nm ionocytes compared with those raised in regular (hypotonic) embryo medium, suggesting that Nm ionocyte induction and differentiation does not depend on water influx caused by differences in osmolarity between the medium and the fish (Fig. 5B). To address whether the increase in Nm ionocyte in DI water (0 mOsmol/l) is caused by a decrease in osmolarity, we supplemented DI water with D-sorbitol and sucrose to embryo medium osmolarity. Nm ionocyte number was comparable with DI water in larvae incubated in these solutions (Fig. 5C). Together, these data demonstrate that Nm ionocyte number is controlled by specific ion concentration, not by media osmolarity.

### Nm ionocyte number is partially dependent on hair cell activity

We showed that the ionocyte induction is ion selective and osmolarity independent. We next asked which cells contribute to Nm ionocyte recruitment. Because Nm ionocytes modulate hair cell function and loss of ionocytes leads to a decrease in hair cell activity (Peloggia et al., 2021; Stawicki et al., 2014), we asked whether hair cells play a role in inducing more Nm ionocytes. To test this, we turned to *pcdh15a* mutants, which develop non-functional hair cells (Fig. 5D) (Seiler et al., 2005). Our analysis shows that mutants have an increased number of mature Nm ionocytes irrespective of whether they were raised in embryo medium or DI water (Fig. 5E). This indicates that disruption in hair cell activity plays a role in controlling number or recruitment of new Nm ionocytes. Interestingly, although inhibiting hair cell function recruits more Nm ionocytes, we see a further increase in Nm ionocyte number in mutants incubated in DI water compared with those incubated in embryo medium. This suggests that, even though hair cell function modulates the number of ionocytes recruited into neuromasts, there must be additional triggers of Nm ionocyte recruitment and differentiation.

Loss of calcium homeostasis can impact hair cell function, and our results raise the possibility that Nm ionocyte number is increased after Ca^2+^ depletion due to hair cell dysfunction. To test this, we incubated *pcdh15a* mutants in embryo medium depleted of Ca^2+^. Mutants show an increase in Nm ionocytes in low Ca^2+^ compared with embryo medium (Fig. 5E). This experiment suggests that Ca^2+^ depletion affects ionocyte recruitment in a hair cell-independent way and provides more evidence of additional triggers of Nm ionocyte recruitment.

Sensory neurons that innervate the skin sense other environmental changes such as temperature and oxygen in other organisms, and could be involved in this process (Dhaka et al., 2006; Makhijani et al., 2011, 2017). *adgra2* mutants lack dorsal root ganglia (DRG) and somatosensory neurons (Bostaille et al., 2016; Malmquist et al., 2013; Vanhollebeke et al., 2015) but show no defects in progenitor activation or Nm ionocyte differentiation based on *foxi3a* and *trpv6* HCRs (Fig. S8A,B). Therefore, we conclude that DRG-derived sensory neurons are not involved in sensing salinity and inducing ionocytes. In summary, although hair cell activity controls Nm ionocyte number, additional sensory neuron-independent mechanisms are likely at play.

## DISCUSSION

Physiological adaptation occurs in individuals that are subjected to environmental perturbations during their lifetime. These changes are rapid and reversible, in contrast to natural selection-driven genetic adaption. One example is the adaptive differentiation of ionocyte subtypes within the body in response to fluctuations in water salinity, including Nm ionocytes (Peloggia et al., 2021). In this study, we show that, in response to a decrease in ion concentration, Nm ionocyte precursors, which are derived from basal stem cells of the skin, upregulate the transcription factors *foxi3a* and *foxi3b*, and divide to give rise to a Nm ionocyte pair. Here, we discuss the possible sources of Nm ionocyte progenitors, and their similarities and differences with other tissue-specific ionocytes. We also discuss possible mechanisms that sense environmental salinity and how comparisons between tissue-specific ionocytes may inform us about the evolution of cell types.

### Are Nm ionocyte progenitors a defined subpopulation of cells or are all basal cells competent to respond to environmental changes?

Basal cells are multipotent stem cells that give rise to several cell types in organs, such as the lungs, skin and tastebuds (Barlow and Klein, 2015; Blanpain and Fuchs, 2006; Wu et al., 2022). Basal stem cells are heterogeneous populations and different subtypes or states have different potencies. In tastebuds, only a subpopulation of SOX2^high^ basal cells can differentiate into taste-receptor cells (Shechtman et al., 2023); in the pulmonary epithelium, subtypes of basal stem cell populations give rise to different lineages (Kotton and Morrisey, 2014). In the frog epidermis, basal cells differentiate into multiciliated cells, small secretory cells and ionocytes (Dubaissi and Papalopulu, 2011; Dubaissi et al., 2014), whereas, in zebrafish, basal cells give rise not only to skin and Nm ionocytes, but also to other cell types, such as keratinocytes, mucous cells and Merkel cells (Brown et al., 2023; Lee et al., 2014; Lu et al., 2021). The basal cell heterogeneity in other systems, combined with our observation that only a small population of cells rapidly responds to low salinity, leads us to hypothesize that Nm ionocytes are derived from a small subpopulation of primed basal stem cells. Adult stem cells are maintained through asymmetric cell divisions to prevent the depletion of the stem cell pool throughout life (Dray et al., 2021; Fuchs and Chen, 2013; He et al., 2009; Van Der Flier and Clevers, 2009). We propose that a similar mechanism may act in the zebrafish skin to maintain pre-determined Nm ionocyte precursors (Fig. S9, top right).

### Different mechanisms for sensing salinity

Low salinity leads to the adaptive differentiation of new Nm ionocytes. We also show that disruption of hair cell function leads to an increase in Nm ionocyte number, indicating that hair cell activity is an important regulator of this process. However, because the number of Nm ionocytes increases even further when mutant larvae are incubated in DI water, additional mechanisms or cell types are likely at play. One possibility is that the lack of hair cell function affects the ionic concentration in the neuromast surroundings. In this case, Nm ionocyte increase would not be directly regulated by hair cells, but an indirect consequence of disrupted hair cell function. Another, maybe more likely hypothesis is that basal cells sense ionic concentration directly and trigger new Nm ionocytes in a cell-autonomous manner. In the mammalian lung, for example, TP63-positive airway stem cells sense oxygen concentrations cell-autonomously and differentiate into solitary neuroendocrine cells, which mitigate hypoxic injury (Shivaraju et al., 2021). The finding that Ca^2+^ depletion increases ionocyte number in the absence of functional hair cells in *pcdh15a* mutants also suggests that ions such as Ca^2+^ are acting directly on basal cells. If basal cells themselves sense ion concentrations, they likely do so through ion channels. However, the identity of these channels is still unknown, as the ion channels expressed in mature ionocytes are not present in progenitors.

It is also possible that salinity sensing and Nm ionocyte differentiation are regulated by systemic responses involving the olfactory system, nerves or hormonal regulation. Zebrafish larvae sense NaCl concentrations via the olfactory epithelium, which impacts swimming behavior, but it remains unknown whether the same circuit controls ionocyte number (Herrera et al., 2021). However, in contrast to what we observed in Nm ionocytes, the olfactory system detects only changes in salinity, not absolute levels, suggesting two distinct mechanisms. Sensory neurons in *Drosophila* detect environmental changes such as oxygen availability and, in response, regulate cell fate (Makhijani et al., 2011, 2017). Here, we demonstrate that DRG-derived sensory neurons are not required for the response to low salinity as Nm ionocyte development is normal in *adgra2* mutants. However, Rohon-Beard neurons are still present in *adgra2* mutants and could potentially be involved in sensing environmental changes.

### Ionocyte number is controlled by different mechanisms in the skin and neuromast

We showed that Ca^2+^ depletion leads to formation of new Nm ionocytes through activation of new progenitors. Likewise, lowering the concentrations of Na^+^ and Cl^−^ in the medium leads to an increase in Nm ionocyte number, but to a lesser extent than Ca^2+^ depletion. On the other hand, K^+^ depletion is not sufficient to induce new Nm ionocytes. These results suggest that not all ions play the same role in Nm ionocyte recruitment. Interestingly, the increase of Nm ionocytes in each condition did not correlate with the ion concentration in normal embryo medium: the concentrations of Na^+^ and Cl^−^ are more than tenfold those of Ca^2+^ and K^+^, but Ca^2+^ removal leads to the strongest response.

Ca^2+^ depletion induces ionocyte proliferation in both skin and neuromasts (Dai et al., 2014; Li et al., 2021; Xin et al., 2019). In neuromasts, low Ca^2+^ leads to formation of a new pair of Nm ionocytes through division of a progenitor cell, which contrasts with the skin, where most NaR ionocytes are solitary. Skin NaR ionocytes remain quiescent during homeostasis due to intracellular Ca^2+^-dependent suppression of PI3K, and this intracellular Ca^2+^ is imported to the cell via the Trpv6 channel (Xin et al., 2019). Under low Ca^2+^ conditions, NaR ionocytes exit quiescence and proliferate (Li et al., 2021; Liu et al., 2018; Xin et al., 2019, 2021). Although Nm ionocyte progenitors also proliferate under low Ca^2+^, they do not express *trpv6*. Mature Nm ionocytes, on the other hand, express *trpv6* but are post-mitotic. These observations suggest that, although low Ca^2+^ induces more ionocytes in both skin and neuromasts, it does so through different mechanisms.

### Notch signaling dynamics differ between skin and Nm ionocyte development

Notch signaling governs the specification and differentiation of several types of ionocyte in different organisms, from fish and frogs to mice and humans (Dubaissi and Papalopulu, 2011; Hsiao et al., 2007; Jänicke et al., 2007; Plasschaert et al., 2018). In the frog epidermis, Notch inhibition leads to an increase in ionocyte number, similar to the zebrafish embryonic skin (Quigley et al., 2011). In the kidneys of mammals, Notch signaling also specifies ion-regulating cells (Jeong et al., 2009; Mukherjee et al., 2019). We show that the Nm ionocyte pair comprises a cell with low Notch signaling (*dll4* positive) and a cell with high Notch signaling (*notch1b* positive), and that Notch signaling is sustained in mature fully differentiated ionocytes (Fig. 2) (Peloggia et al., 2021). This contrasts with skin ionocyte differentiation in fishes and frogs, where Notch signaling is active at the onset of fate determination but is not maintained in mature cells. In the embryonic zebrafish skin, Notch-dependent lateral inhibition leads some basal stem cells to express *dlc* and *jagged* ligands, surrounded by cells that express *notch1a* and *notch3* receptors (Hsiao et al., 2007; Jänicke et al., 2007). This results in the differentiation of the low Notch cell (*dlc* positive) into a skin ionocyte, whereas the Notch-positive cell becomes a keratinocyte (Fig. S9, left panel).

The different roles played by Notch signaling in skin and Nm ionocyte development are apparent in loss- and gain-of-function experiments. Global downregulation of Notch signaling in *mindbomb* mutants or pharmacological Notch inhibition lead to an increase in skin ionocyte density at expense of keratinocytes (Chen et al., 2019; Hsiao et al., 2007). In contrast, pharmacological inhibition or loss of Notch signaling in *dll4* mutants causes loss of mature Nm ionocytes, but no changes in the progenitor population (Fig. 3) (Peloggia et al., 2021). These results demonstrate that although Notch signaling is crucial for proper ionocyte specification, differentiation or survival, the roles played by Notch are context and tissue dependent.

Although *dll4* plays a pivotal role in Nm ionocyte survival, it has not been shown to have a role in skin ionocytes. Here, we show that *dll4* mutants lack the NaR ionocyte population in the skin, concomitant with an expansion of the HR population. These two subtypes of ionocytes share a common ionocyte progenitor (Fig. S9), and little is known about how this progenitor decides between the NaR or HR fate. It has been suggested that *foxi3b* levels drive differentiation of each subtype, but this has not been demonstrated (Hsiao et al., 2007). Our results suggest that *dll4* may play a role in this decision, and its loss shifts the population entirely to the HR fate. An alternative possibility is that, similarly to Nm ionocytes, NaR ionocytes also undergo cell death in the mutants, and the HR population increases to compensate for their loss.

### Tissue-specific ionocytes in physiology and disease

Ion-regulating cells exist in many invertebrate and vertebrate species (Dubaissi and Papalopulu, 2011; Guh et al., 2015; Henry et al., 2012; Leguen et al., 2015; Mauduit et al., 2022; Montoro et al., 2018; Pou Casellas et al., 2023; Sakamoto et al., 2015). However, through the years, ion-regulating cells have received diverse names in different organs and in different species, making comparisons between cell types challenging. Although homology between these cell types has not been established, mammalian ionocytes express the *foxi3a/b* ortholog Foxi1. Likewise, hemichordates (acorn worms) and cephalochordates (amphioxi) possess Foxi1-positive cells in their gills, which are hypothesized to be proto-ionocytes (Sackville et al., 2022). These putative ionocytes express several of the ion channels present in zebrafish ionocytes. Therefore, appearance of bona fide ionocytes likely pre-dates the evolution of vertebrates and of a lateral line system. The emergence of tissue-specific ionocytes in vertebrates serves as an excellent new model for understanding the evolution of cell type diversification (Arendt et al., 2016).

As mentioned above, mammalian ionocytes are defined as Foxi1^+^ cells that contribute to ionic homeostasis of the organ in which they reside (Shah et al., 2022). However, before molecular data were available, these cells residing in tissues such as the kidney, inner ear, prostate, epididymis, thymus and skin were identified based on functional analyses or morphological characteristics, such as high mitochondrial content and presence of apical microvilli (Pou Casellas et al., 2023). Recently, ionocytes have also been identified in mammalian salivary glands and lungs (Mauduit et al., 2022; Montoro et al., 2018; Plasschaert et al., 2018). In the lung, ionocytes express the ion channel CFTR, frequently mutated in individuals with cystic fibrosis and FOXI1 loss significantly decreases expression of CFTR in the mouse airway epithelium and leads to cystic fibrosis-like phenotypes (Montoro et al., 2018). In the mammalian inner ear, mutations in the HCO3^−^/Cl^−^ transporter pendrin lead to hearing loss caused by endolymph acidification (Everett et al., 1997; Nakaya et al., 2007; Wangemann et al., 2007). In teleost ears, ionocytes have also been suggested to regulate ion homeostasis of the endolymph (Becerra and Anadón, 1993; Mayer-Gostan et al., 1997). However, although much work has focused on the consequences of ionocyte loss of function, less is known about their lineage and dynamics in mammalian systems. It would be valuable to determine whether the adaptive regulation of ionocyte number is unique to open systems, such as the lateral line, or whether it also occurs in closed organs, such as the inner ear.

### Ionocytes, adaptability and evolution

Nm ionocyte recruitment is controlled by environmental changes, such as fluctuations in salinity and pH, where they likely regulate hair cell function (Peloggia et al., 2021). We propose that Nm ionocyte number is an example of phenotypic plasticity that increases fitness in different environments. Development exhibits remarkable plasticity, and although some environmental changes may not lead to significant changes in the overall observable phenotype, our study shows instances where cellular and tissue adaptations are crucial for maintaining fitness. These changes can be easily overlooked, and often evade detection by bulk techniques or are even considered experimental noise. In some cases, because we lack understanding of which external stimuli are sensed, these parameters are challenging to control in the laboratory setting. Nm ionocytes represent a simple and amenable model to test how the external environment affects cell state, differentiation and organ homeostasis.

Seasonal environmental fluctuations profoundly shape the development and physiology of life on Earth. Plants, animals and even bacteria integrate cues of seasonality to anticipate and properly respond to environmental changes (Fretwell, 1972). Looking ahead, especially amid anthropogenic actions leading to dramatic changes in the environment – such as climate change – these adaptive responses may be essential for the survival of organisms.

## MATERIALS AND METHODS

### Zebrafish lines and husbandry

All experiments were performed following the guidelines of the Stowers Institute IACUC review board. Embryos were raised in 0.5× embryo E2 medium [7.5 mM NaCl, 0.25 mM KCl, 0.5 mM MgSO_4_, 75 mM KH_2_PO_4_, 25 mM Na_2_HPO_4_, 0.5 mM CaCl_2_, 0.5 mg/l NaHCO_3_ (pH 7.4)] unless specified otherwise. Wild-type fish used were either AB or TU. The following published transgenic lines were used in this study: *Tg(-8.0cldnb:lynEGFP)^zf106Tg^* (Haas and Gilmour, 2006), *Tg(-8.0cldnb:H2A-mCherry)^psi4Tg^* (Romero-Carvajal et al., 2015), *Tg(tp1bglobin:EGFP)^um14^* (Parsons et al., 2009) and *Tg(she:H2B-EGFP)^psi59Tg^* (Peloggia et al., 2021).

### Mutant line generation

Mutants for *dld* and *dll4* genes were generated using CRISPR/Cas12a*.* For the *dll4^psi87^* mutation, two gRNAs targeting sequences 869 bp upstream of the translation start site (5′-GTATCCCAGAAAACACTATA-3′) and in exon 4 (5′-GTTGCAGATGAACCGGTAAG-3′) were designed using DeepCpf1 (Luo et al., 2019) and checked for off-target effects using CRISPRscan (Moreno-Mateos et al., 2017). For the *dld^psi86^* mutant, two CRISPR/Cas12a guides were designed using the same parameters and tools, and targeted regions 382 bp upstream of the translation start site (5′-GATCATCTCCACATGAACTT-3′) and 215 bp downstream of the stop codon (5′-CCAGGAACGTGCCAAATGGA-3′).

For both mutants, one-cell stage zebrafish embryos were injected with 50 nM EnGen Lba Cas12a (Cpf1) (New England Biolabs) and each gRNA at a final concentration of 2 nM. Injected larvae were raised to adulthood and germline transmission of the mutation was confirmed by PCR using the following primer pairs: *dll4*-FwA, 5′-TTTGGGATTCACCTTGAACG-3′; *dll4*-FwB, 5′-GCCTGGCACTCACCTTACTC-3′; *dll4*-Rv, 5′-ATAACTGGCCATCTGGGTTG-3′; *dld*-Fw, 5′-GCCTGTATATAACCGGCTGC-3′; *dld*-RvA, 5′-GTCAGTGCATATGCATACACAG-3′; and *dld*-RvB, 5′-TCATTAGTCGTCCCATGGCG-3′.

Once F0 founders were identified, one founder per gene was outcrossed to wild-type fish to generate a stable F1 generation. The F1 generations were then raised to adulthood and heterozygous carriers of the deletion were identified by genotyping PCR from fin clips. Experiments were performed with embryos derived from incrosses of either F1 or F2 parents.

### Transgenic line generation

The *Tg(dld:hist2h2l-EGFP)^psi84Tg^* knock-in was generated via CRISPR-knock-in based on a previously published method (Kimura et al., 2014) with modifications for Cas12a. The Gbait vector was first modified via replacement of the Gal4FF-BGH-polyA-Kanamycin resistance sequence (BamHI/SalI digested) with a *hist2h2l-EGFP-sv40* polyA fragment (BglII/XhoI digested)*.*

A Cas12a targeting sequence, 5′-ACACAGGAAACAGCTATGAC-3′, was identified within the GBait plasmid upstream of the T3 primer sequence. This sequence is not predicted to target the zebrafish genome. We verified that this guide RNA digested the Gbait plasmid in combination with Lba Cas12a (New England Biolabs) in an *in vitro* reaction. To identify gene specific guides, DeepCpf1 (Luo et al., 2019) was used, followed by BLAST of the zebrafish genome to ensure specificity. A guide (5′-GAGATGAAAACTTCAAACT-3′) was identified that would target 940 bp upstream of the 5′UTR of *dld*. All guide RNAs were ordered from IDT, then diluted to a stock concentration of 24 µM (Fernandez et al., 2018).

To make the injection mix, the Gbait and *dld* gRNAs were combined at 4.8 µM each with 20 µM Lba Cas12a protein (New England Biolabs) and incubated for 10 min at 37°C. The mix was then placed on ice and 30 ng of Gbait-H2B-GFP plasmid and Phenol Red were added. We injected 1-3 nl of the mix into the cell of one-cell stage zebrafish embryos. Embryos were raised at 28°C then screened for GFP expression at 1-2 dpf. GFP-positive larvae were raised to adulthood then screened for germline transmission of GFP.

### Hybridization chain reaction

Hybridization chain reaction (HCR) was adapted from the manufacturer's instructions (Choi et al., 2018) (Molecular Instruments). Embryos were fixed overnight with 4% PFA at 4°C, then dehydrated in an ethanol series up to 100% ethanol. Dehydrated samples were stored at −20°C from overnight up to 6 months. Samples were then rehydrated and washed twice with H_2_O+0.1% Tween20 (Sigma-Aldrich) and incubated in 80% acetone at −20°C for 20 min. The samples were then washed twice in room temperature PBST (PBS+0.1% Tween20) and incubated in 200 µl of Hybridization Buffer (Molecular Instruments) for 30 min at 37°C. Later, samples were incubated in Hybridization Buffer with 2 pmol of each probe at 37°C overnight. On the next day, samples were washed four times for 15 min each with pre-warmed Washing Buffer (Molecular Instruments) and twice in 5×SSCT for 5 min, and subsequently incubated for at least 30 min with 200 µl of Amplification Buffer equilibrated at room temperature. Finally, samples were incubated overnight with the suitable amplifiers for each experiment, then washed three times with 5×SSCT and incubated with DAPI (1 µg/ml). Samples were protected from light and at 4°C before imaging.

The following probes were used in this study: *notch1a*-B2, *notch1b*-B2, *notch3*-B2, *dla*-B1, *dlb*-B1, *dlc*-B1, *dld*-B5, *dll4*-B1, *jag1a*-B1, *jag1b*-B1, *jag2a*-B1, *jag2b*-B1, *her15*-B2, *hes6*-B1, *her4*-B2, *hey1*-B4, *lfng*-B2, *foxi3a*-B2, *foxi3b*-B4, *trpv6*-B1, *fosab*-B4, *junba*-B1, *dusp1*-B2, *kcnj1a*-B2, *rhcgb*-B5, *slc4a1b*-B1 and *egfp*-B2. The *her4*, *kcnj1a* and *her15* probes do not distinguish between tandem duplicates due to sequence similarity. Amplifiers were conjugated either with Alexa488, Alexa546 or Alexa647.

### Immunohistochemistry

Zebrafish larvae at different stages were anesthetized with MS222 (160 mg/ml) and fixed overnight at 4°C with 4% PFA. Standard antibody staining was performed for a5 (DSHB, AB_2166869) (Bradford et al., 2022).

### Cell proliferation analysis

Larvae were treated for 24 h with 3.3 mM EdU (Carbosynth) with 1% DMSO in E2, then fixed in 4% paraformaldehyde overnight at 4°C. Larvae were then stained for HCR and with EdU simultaneously (Ćorić et al., 2023). Briefly, the HCR protocol was performed as described above, until the probe wash step. EdU was then clicked with 2 mM CuSO4, 0.4% Triton-X, 2.5 µM 594-Azide and 50 mM ascorbic acid for 30 min and washed three times for 10 min with PBT (PBS+0.8% Triton-X). After the EdU developing reaction, samples were incubated in HCR amplification buffer (Molecular Instruments) for 30 min and incubated overnight at room temperature with B4-647 amplifier.

### Acclimation of zebrafish larvae to different salinities and media

For acclimation in different media, fish were raised in embryo medium at a density of 40 embryos per dish until 3 dpf, with daily media changes. After reaching 3 dpf, fish were incubated in fresh, regular or modified embryo medium. For calcium depletion, embryo medium was made without CaCl_2_; for potassium depletion, embryo medium was made without KCl and KH_2_PO_4_; for sodium and chloride depletion, no NaCl, Na_2_HPO_4_ or NaHCO_3_ were added to the media. Isotonic solutions (270 mOsmol/l) were made with either 242 mM sorbitol or 238 mM sucrose. Media pH was always adjusted to 7.2 on the same day of use. Fish were incubated in different media for 48 h at 28.5°C unless specified otherwise.

### Quantification of Nm ionocytes

Mature Nm ionocytes were identified and counted with double HCRs for *foxi3a* and *trpv6*, while progenitors were considered as *foxi3a*-positive and *trpv6-*negative cells adjacent to neuromasts and with a crescent morphology. Neuromasts from the anterior lateral line (Ml1, O2, Ml2, IO4 and O1) and posterior lateral line (L1-L3, LII.1 and LII.2) were quantified (Ghysen and Dambly-Chaudière, 2007; Hwang and Chou, 2013). Numbers were averaged per larvae and displayed as one value that could also be lower than 1 if not all neuromasts contained ionocytes. For ionocyte frequency quantifications, neuromasts were classified based on the presence and absence of ionocytes, and data are displayed as percentage of neuromasts that contained ionocytes. In some experiments (Figs 3F, 5A and 5C), data between replicates have a wide distribution, and comparison between replicates was challenging. In these cases, data were normalized by the average of the embryo medium control of each replicate. Normalized data are indicated in the respective figure legend.

For Nm ionocyte progenitors, both individual and pairs of *foxi3a/b-*expressing cells were counted as one progenitor unit. Cells were also only considered to be progenitors when they were immediately adjacent to neuromasts and not expressing the ion channel *trpv6*. When pairs were already expressing *foxi3a* and *trpv6* and were fixed while invading neuromasts, they were considered to be mature ionocytes.

### Time-lapse and confocal imaging

Images were acquired on a Nikon Ti2 Yokogawa CSU-W1 spinning disk head equipped with a Hamamatsu Orca Fusion sCMOS. Objective lenses used were CFI Apo LWD 40× WI 1.15 NA Lambda S and CFI Apo 20× WI 0.95 NA Lambda S.

For live imaging experiments, larvae were slowly anesthetized with tricaine (MS222) up to 150 mg/l and mounted in glass-bottomed dishes (Cellvis) using 0.8% low melting point agarose dissolved in either embryo medium or system water supplemented with MS222 (120 mg/l) (Venero Galanternik et al., 2016). A Stage Top Incubator (OkoLab) was used to keep the constant temperature of 28.5°C and 85% humidity.

For live skin ionocyte labeling, 32 hpf *cldnb:lynEGFP* transgenic larvae were treated with a 30 min 400 nM MitoTracker Red FM (Invitrogen) pulse. Early treatment avoids labeling of lateral line hair cells, as those are not yet functional. Larvae were then kept in the dark until 3dpf, when they were incubated in DI water and imaged as specified above.

A Nikon LUNV solid state laser launch was used for lasers 395/405, 488, 561 and 647 nm for DAPI, GFP/Alexa488, RFP/Alexa546 and Alexa647, respectively. Emission filters used on the Nikon were 480/30, 535/30 and 605/52. Nikon Elements Advanced Research v5.41.02 (Nikon) was used for image acquisition.

### Statistical analysis

All statistical tests and plots were performed in GraphPad Prism 10 (version 10.0.2). *P-*values smaller than 0.05 were considered significant. Normal distribution was assessed using a Kolmogorov–Smirnov test. Data from two groups were compared using a two-tailed unpaired *t*-test or a two-tailed Mann–Whitney *U*-test. When comparing data from more than two groups, statistical significance was calculated using either one-way ANOVA with Dunn’s multiple comparison test or non-parametric Kruskal–Wallis ANOVA with Dunn's multiple comparison test. Plots were made in GraphPad Prism 10 or the R package ggplot2 (Wickham, 2011).

### Image analysis

Fluorescence intensity quantification of transgenic lines and HCRs (*dusp1*, *fosab* and *junba* HCRs) were performed in Fiji (Schindelin et al., 2012). Images were *z*-projected (maximum intensity projection), background was removed with the ‘Subtract Background’ function, and a rolling ball radius of 50 pixels was used for all images and measured channels. Then, a region of interest was drawn around the cell or cells of interest and measured using the ‘Measure’ function. Values were then exported and the mean fluorescence intensity per ROI was imported into GraphPad Prism 10 for statistical analysis and graphing.

To determine expression of HCR probes at the single cell level in mature ionocytes, we used the Notch reporter *Tg(tp1bglobin:EGFP)^um14^*, which is expressed only in the NaR-like Nm ionocyte. For progenitors, we observed that *foxi3b* is expressed in the progenitor before cell division and in both mature Nm ionocytes. For progenitors, we observed that *foxi3b* is expressed in the progenitor cell before cell division, in the two daughter cells and subsequently in both mature Nm ionocytes. Therefore, for all analyses of progenitor cells, we incorporated *foxi3b* to identify progenitor cells before and after division. For the experiments in Fig. 2B,D,E, where expression of candidate genes is evaluated within the pair, we only analyzed expression that overlapped with the nuclei of *foxi3b^+^* cells to determine cellular resolution.

For HCR quantification of Notch receptors and putative downstream factors, we automatically segmented Notch-positive Nm ionocytes using the *Tg(tp1bglobin:EGFP)^um14^* transgenic line. To segment the ionocytes, the image file was opened with the python package tifffile (Gohlke, 2023) and the ionocyte channel was preprocessed with background subtractions and smoothing. A 3D white tophat (scipy) (Gommers, 2023) with a footprint of size of 50×50×3 pixels was used for background subtraction, and a 3D gaussian filter with a sigma of 1×1×0.5 was used for smoothing. After preprocessing, a threshold was applied to the image at a grey value of 500 to produce a segmentation mask. To slightly reduce the width of each mask, an Euclidian distance transform was calculated and pixels with transform values less than 2 were set to zero, whereas pixels with value 2 or greater were set to one. The resulting mask will often have multiple parts that merge into one structure at the bottom of the *z*-stack, as the Notch reporter also labels central support cells in the neuromast. To divide and segment individual cells, the *z*-stack was treated as a timelapse, and each cell was tracked in *z*. Individual cells were determined by only using trajectories before merge events. With the desired cells segmented, the other channels were background subtracted by white tophat with a footprint of (15, 15, 1) and the mean intensity within each cell was measured.

Quantification of fluorescence in either the *dld:H2B-EGFP* or asymmetry in *dld* expression via HCR was carried out in Fiji. In these cases, either the brightest slice or a maximum intensity projection of up to three brightest slices was quantified. Background was not subtracted but the comparison was performed pairwise with the cell from the same image.

In the case of some reporter lines, such as the *dld:H2B-EGFP* line, fluorescence intensity is heterogeneous between cells. Therefore, we applied nonlinear changes (gamma) to make all features visible. Gamma ranged from 0.5 to 0.8 and was applied whenever stated in figure legends. Image quantification was always performed in the raw file and never with gamma-modified images.

## Supplementary Material



10.1242/develop.202809_sup1 Supplementary information
