# Supplementary material for: Environmental and molecular control of tissue-specific ionocyte differentiation in zebrafish
Source: Development. 2024 Oct 22;151(20):dev202809. doi: 10.1242/dev.202809 (PMC11528218; doi:10.1242/dev.202809)
Supplement: Supplementary information [file develop-151-202809-s1.pdf]

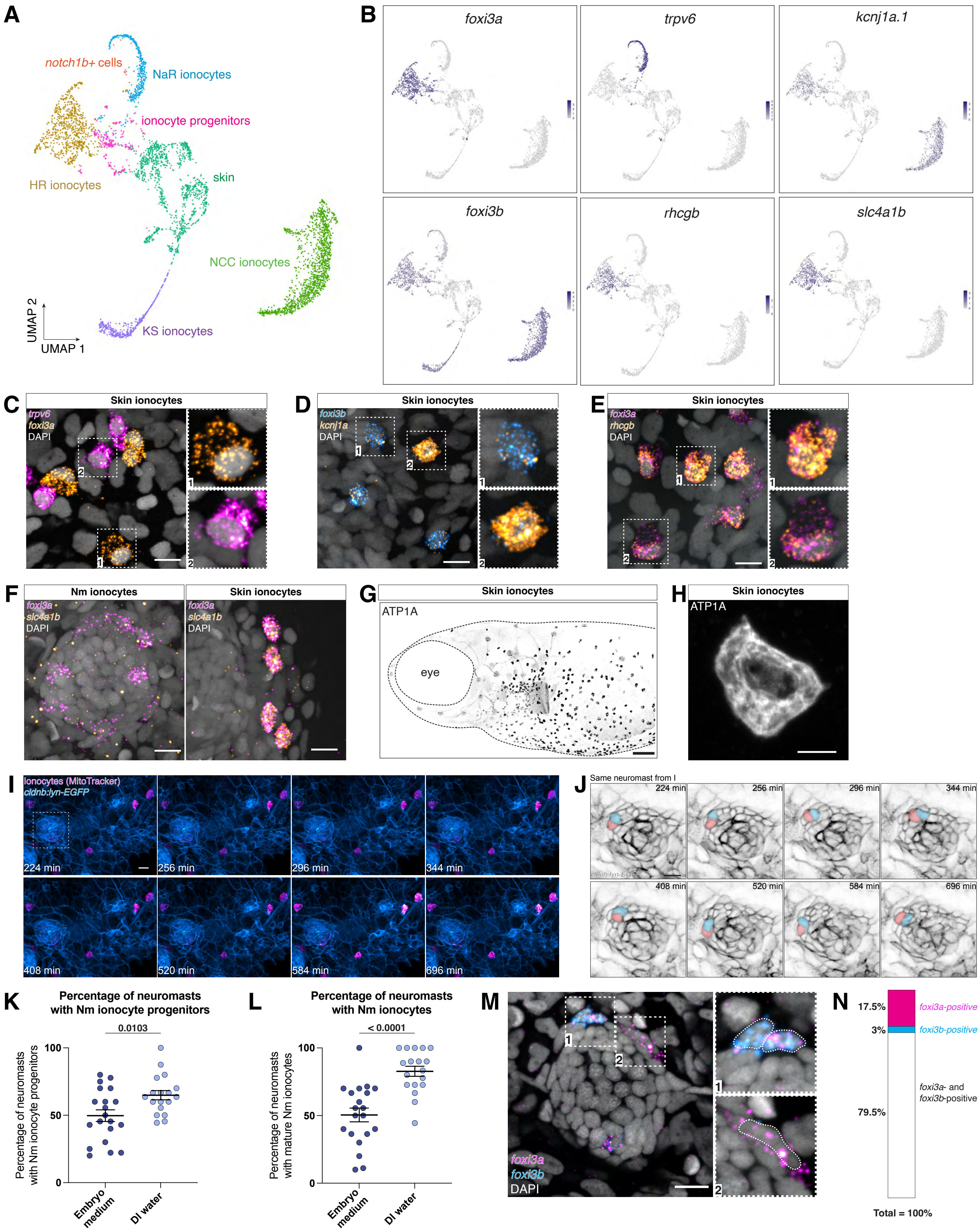
A
notch1b+ cells
NaR ionocytes
ionocyte progenitors
HR ionocytes
skin
NCC ionocytes
KS ionocytes
UMAP 2
UMAP 1
B
foxi3a
trpv6
kcnj1a.1
foxi3b
rhcgb
slc4a1b
C
Skin ionocytes
trpv6
foxi3a
DAPI
D
Skin ionocytes
foxi3b
kcnj1a
DAPI
E
Skin ionocytes
foxi3a
rhcgb
DAPI
F
Nm ionocytes
foxi3a
slc4a1b
DAPI
Skin ionocytes
G
Skin ionocytes
ATP1A
eye
H
Skin ionocytes
ATP1A
I
Ionocytes (MitoTracker)
cldnb:lyn-EGFP
224 min
256 min
296 min
344 min
408 min
520 min
584 min
696 min
J
Same neuromast from I
K
Percentage of neuromasts with Nm ionocyte progenitors
0.0103
Percentage of neuromasts with Nm ionocyte progenitors
100
50
0
Embryo medium
DI water
L
Percentage of neuromasts with Nm ionocytes
< 0.0001
Percentage of neuromasts with mature Nm ionocytes
M
foxi3a
foxi3b
DAPI
N
17.5%
3%
79.5%
foxi3a-positive
foxi3b-positive
foxi3a- and foxi3b-positive
Total = 100%

Fig. S1. Differences in gene expression and morphology between skin and Nm ionocytes

(A) UMAP showing different clusters of ionocyte subtypes from Peloggia and Muench et al., 2021. (B) Feature plots for markers of different ionocyte subtypes (C) Maximum intensity projection of skin HR ionocytes, labeled by *foxi3a*, and NaR ionocytes, labeled by *trpv6.* (D) Some skin ionocytes, labeled by *foxi3b* HCR, express *kcnj1a* (E) Some skin ionocytes express *rhcgb*. (F) Nm ionocytes, mature or progenitors (left), do not express *slc4a1b*, but skin ionocytes do (right). (G) Whole mount 5 dpf zebrafish larva in which ionocytes are stained with Na(+)/K(+) antibody. (H) High magnification image of (G) depicting skin ionocyte morphology. Scale bar = 5 μm. (I) Still

images from time lapse showing skin ionocytes labeled with MitoTracker. (J) Zoomed in neuromast from (I) shows a MitoTracker-negative progenitor pair invading a neuromast (K) Frequency of Nm ionocyte progenitors in different media depicted as the percentage of neuromasts in a larva that contain one or more Nm ionocyte progenitors (n = 18 larvae, 170 neuromasts; unpaired t-test). (L) Similar to analysis as in (K) for mature Nm ionocytes (unpaired t-test). (M) Neuromast containing two sets of Nm ionocyte progenitors. In “1”, both *foxi3a* and *foxi3b* are expressed in both cells of the progenitor pair, while in “2” the progenitor is a single cell expressing only *foxi3a*. (N) Percentage of cells that co-express both transcription factors, *foxi3a* and *foxi3b*, and of cells that express only one out of the two factors. Scale bars = 10 μm unless specified otherwise.

Development • Supplementary information

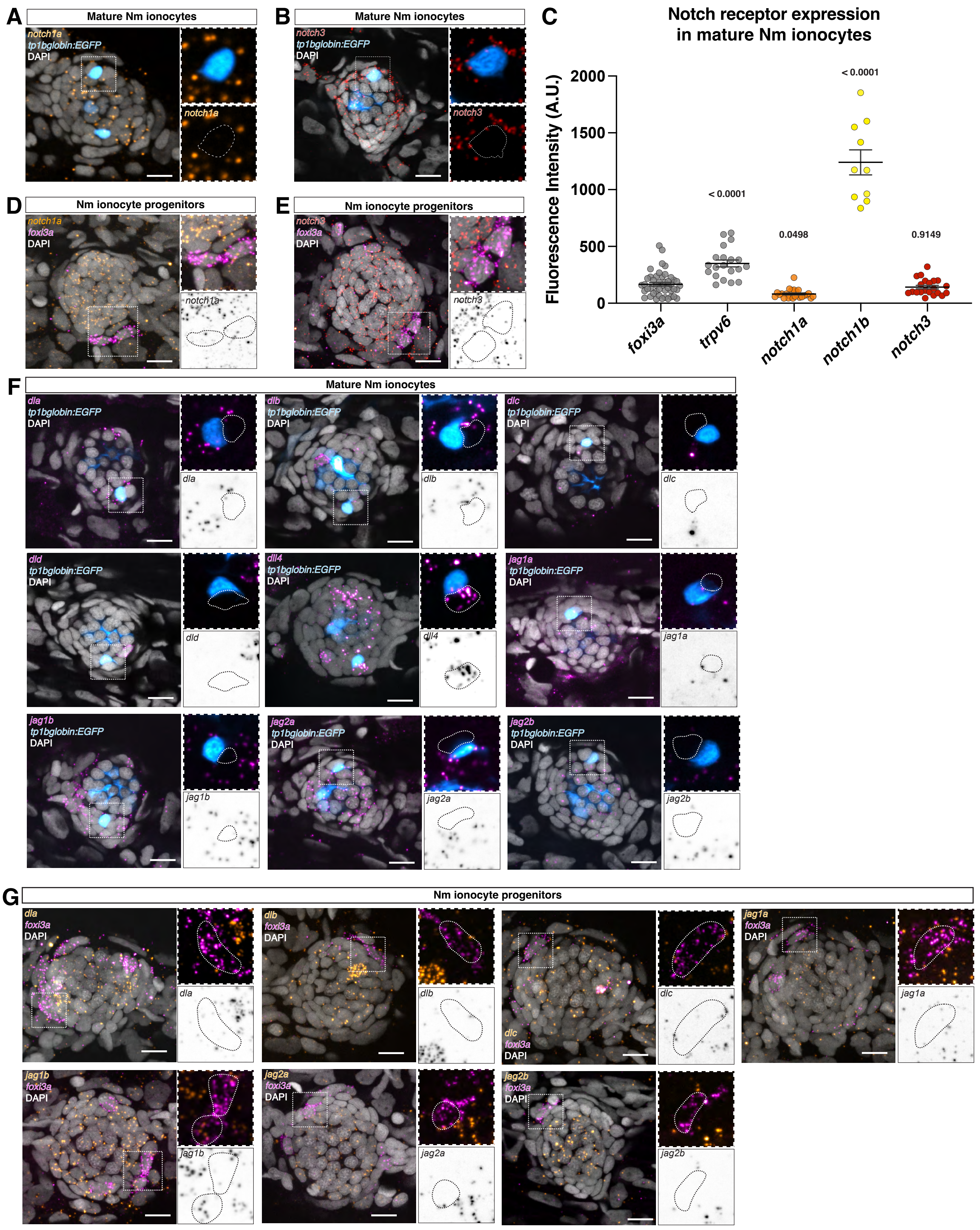

Fig. S2. Notch ligand and receptor expression in Nm ionocytes

(A) HCRs for *notch1a* and (B) *notch3* in mature ionocytes. (C) Quantification of A, B and Fig. 2A. *foxi3a* HCR serves as a negative control and *trpv6* HCR is used as a positive control of signal in Notch reporter-positive cells. Quantification was performed using the Notch reporter channel as a segmentation mask (see Methods) (D) Lack of expression of *notch1a* and (E) *notch3* in Nm ionocyte progenitors. (F) HCR for all other Notch ligands present in zebrafish in mature and (G) Nm ionocyte progenitors. Scale bars = 10 μm.

Development • Supplementary information

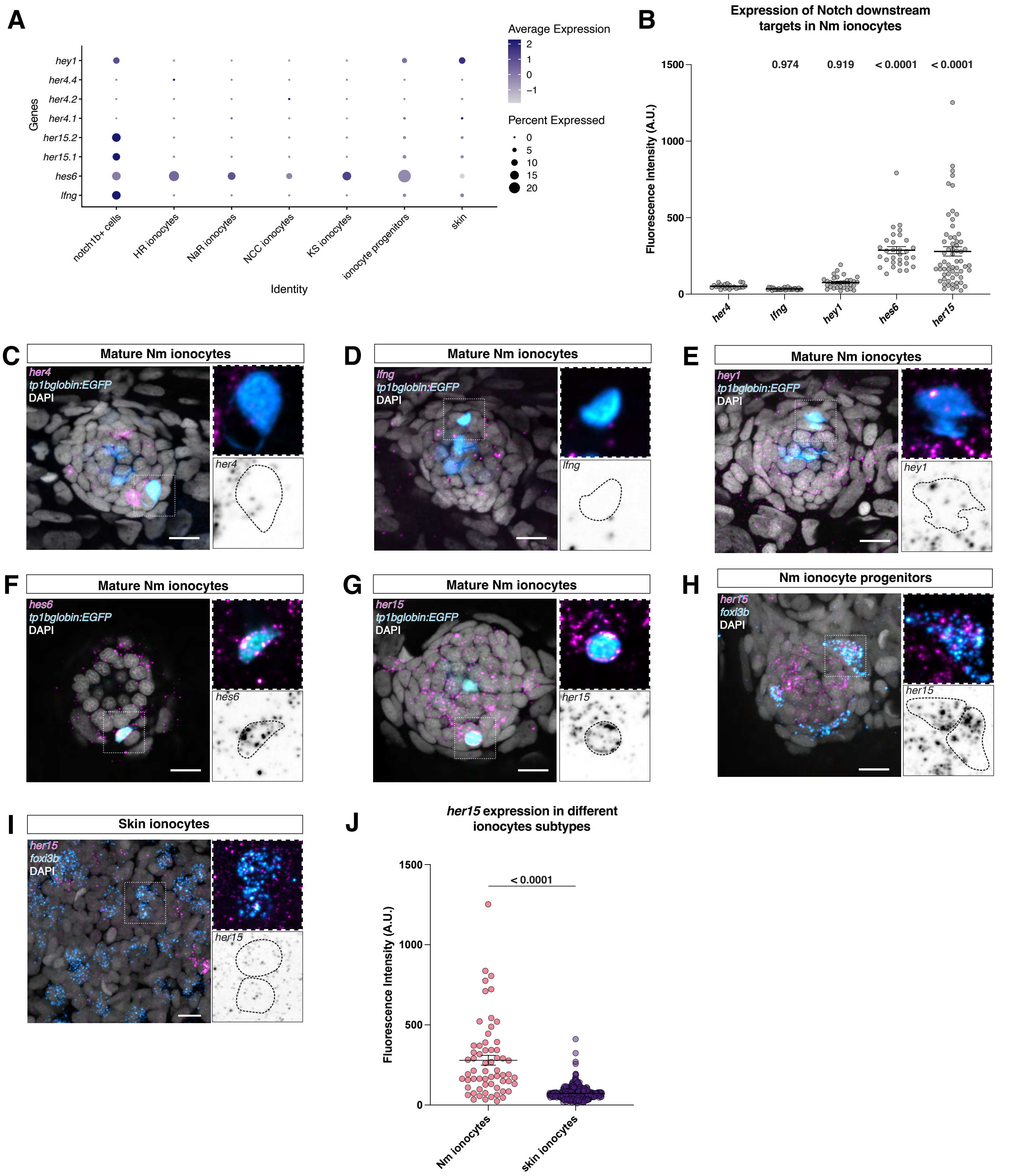

Fig. S3. Analysis of Notch downstream target genes in Nm ionocytes reveals putative Nm ionocyte specific targets

(A) Dot plot from clusters on Figure S1A depicting candidate Notch downstream factors present in Nm ionocytes (*notch1b+* cells). (B) Quantification of panels (C-G), which show expression of Notch targets in mature Nm ionocytes. *her4* is used as a negative control, as it is not detected in the scRNA-seq data (C) HCRs for *her4*, (D) *lfng*, (E) *hey1*, (F) *hes6*, and (G) *her15*. (H) *her15* expression in Nm ionocyte progenitors and (I) in the skin. (J) Quantification of *her15* expression in skin ionocytes when compared to Nm ionocytes (Nm ionocyte data from (B)). Scale bars = 10 μm.

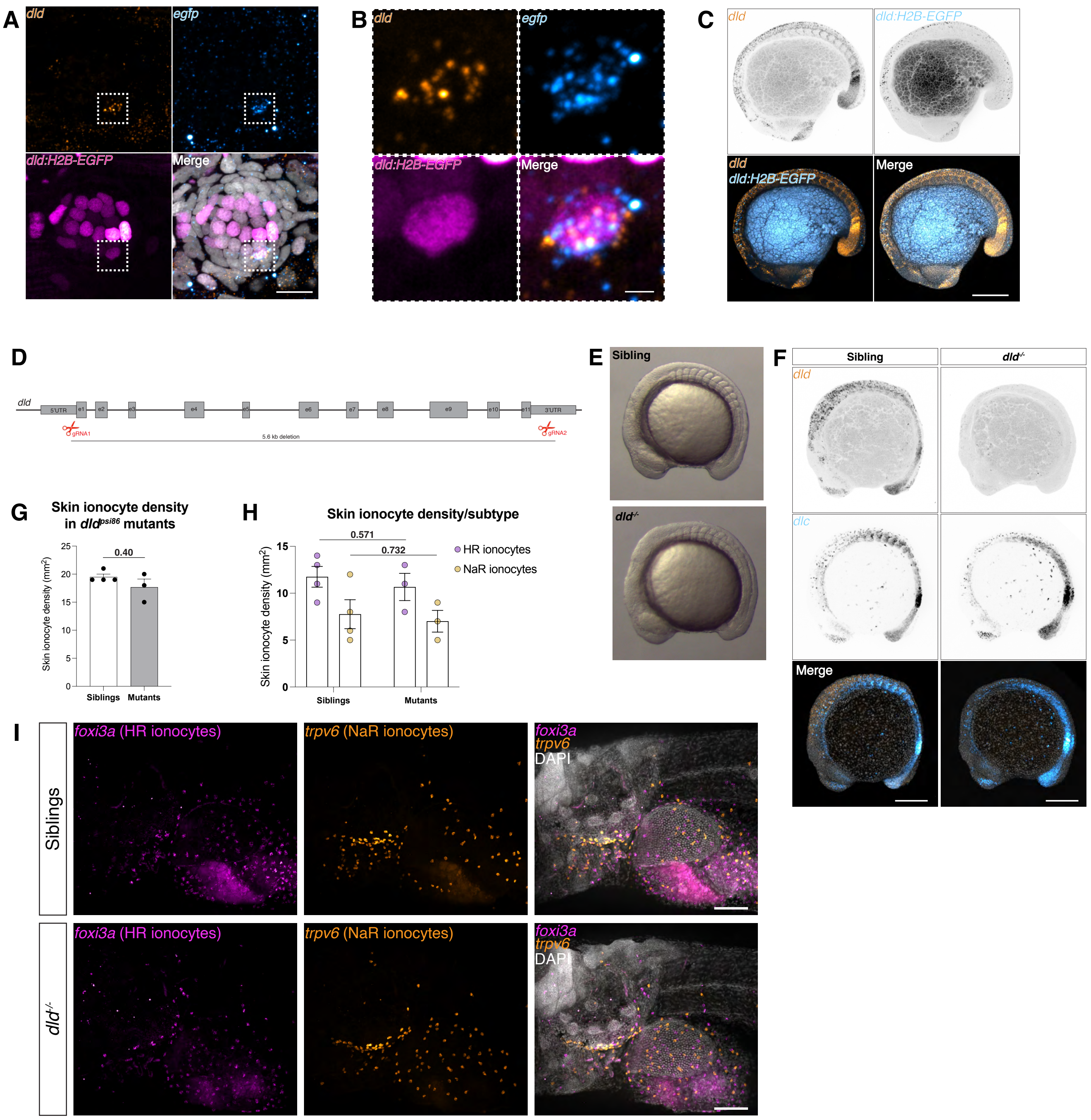

Fig. S4. *dld* mutants do not have any effects on ionocyte development and homeostasis (A) Maximum intensity projection of a neuromast from *dld:H2B-EGFP* fish and stained for the endogenous *dld* and *egfp* transcripts at 5dpf, scale bar = 10 μm. (B) Zoom in of positive hair cell progenitor, scale bar = 2 μm. (C) *dld:H2B-EGFP* zebrafish embryo at 16 hpf showing overlap of endogenous *dld* transcript and EGFP protein in the whole embryo, scale bar = 250 μm. (D) Schematic of *dld* gene deletion strategy using CRISPR/Cas12a. (E) Bright field images of 10hpf sibling and *dld* mutants show somite phenotype in mutants (F) HCR staining for *dld* and *dlc* transcripts show specific downregulation of *dld* transcripts in *dld* mutants, scale bar = 200 μm. (G) Quantification of total skin ionocyte density in the yolk sac region of 5 dpf zebrafish larvae between siblings and mutants (Unpaired t-test) (H) Data from (G) split into HR and NaR skin ionocytes. (I) Representative images from (H), scale bar = 100 μm.

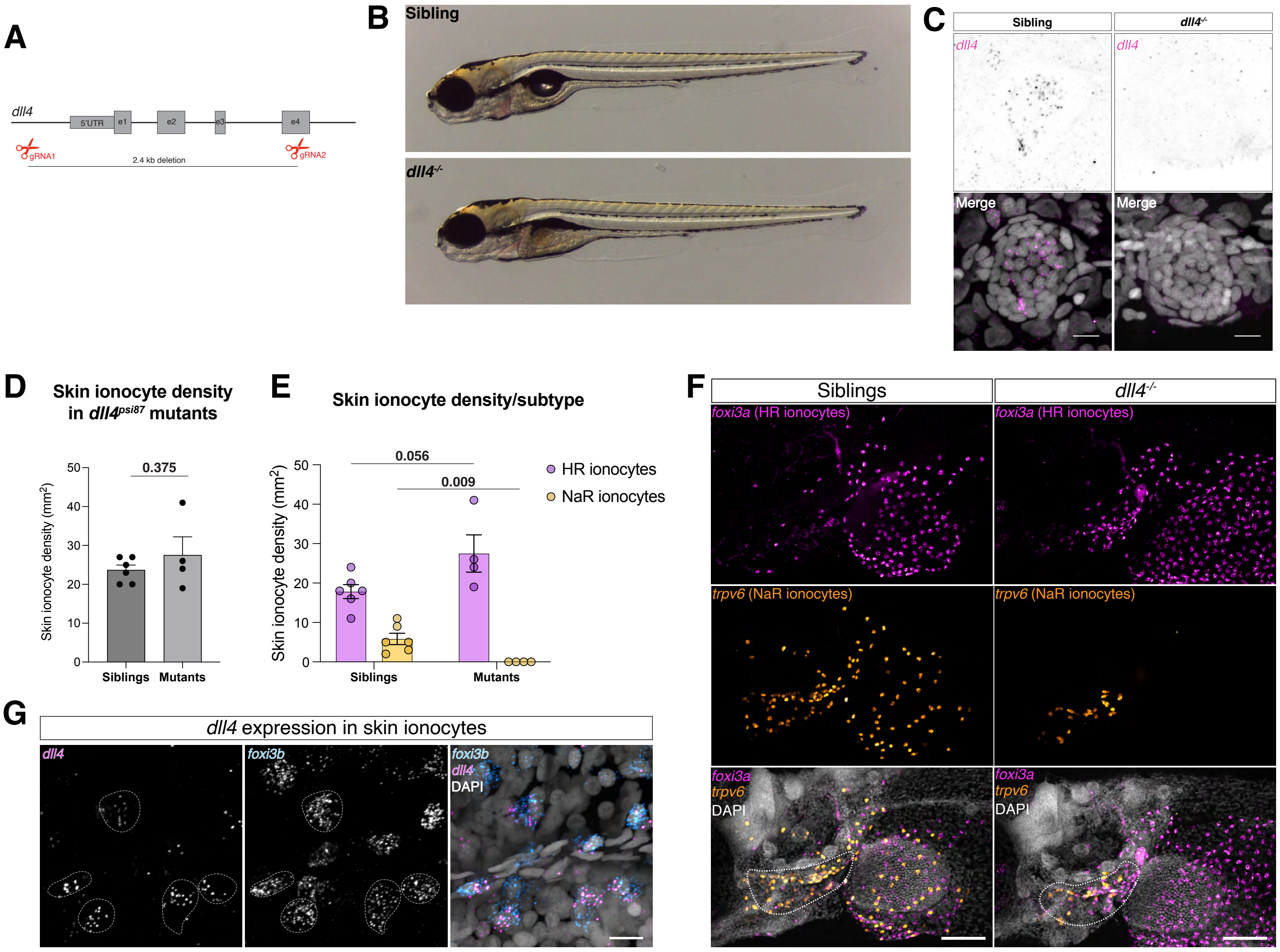

Fig. S5. Manipulation of the Notch pathway shows a role for *dll4* in ionocyte development

(A) Schematic of *dll4* gene deletion strategy using CRISPR/Cas12a. (B) Bright field images of 5 dpf sibling and *dll4* mutants show lack of inflated swim bladder in mutants (C) HCR for *dll4* in 5 dpf fish shows lack of transcript in hair cell of mutants, scale bar = 10 μm (D) Quantification of total skin ionocyte density in the yolk sac region of 5 dpf zebrafish larvae (Unpaired t-test) (E) Data from (D) split into HR and NaR ionocyte subtypes (Unpaired t-test). (F) Representative images from, scale bar = 10 μm (C). Gills are outlined in dashed white. (G) HCR for *dll4* and *foxi3b* in 5 dpf zebrafish larvae shows expression of *dll4* in skin ionocytes, scale bar = 100 μm.

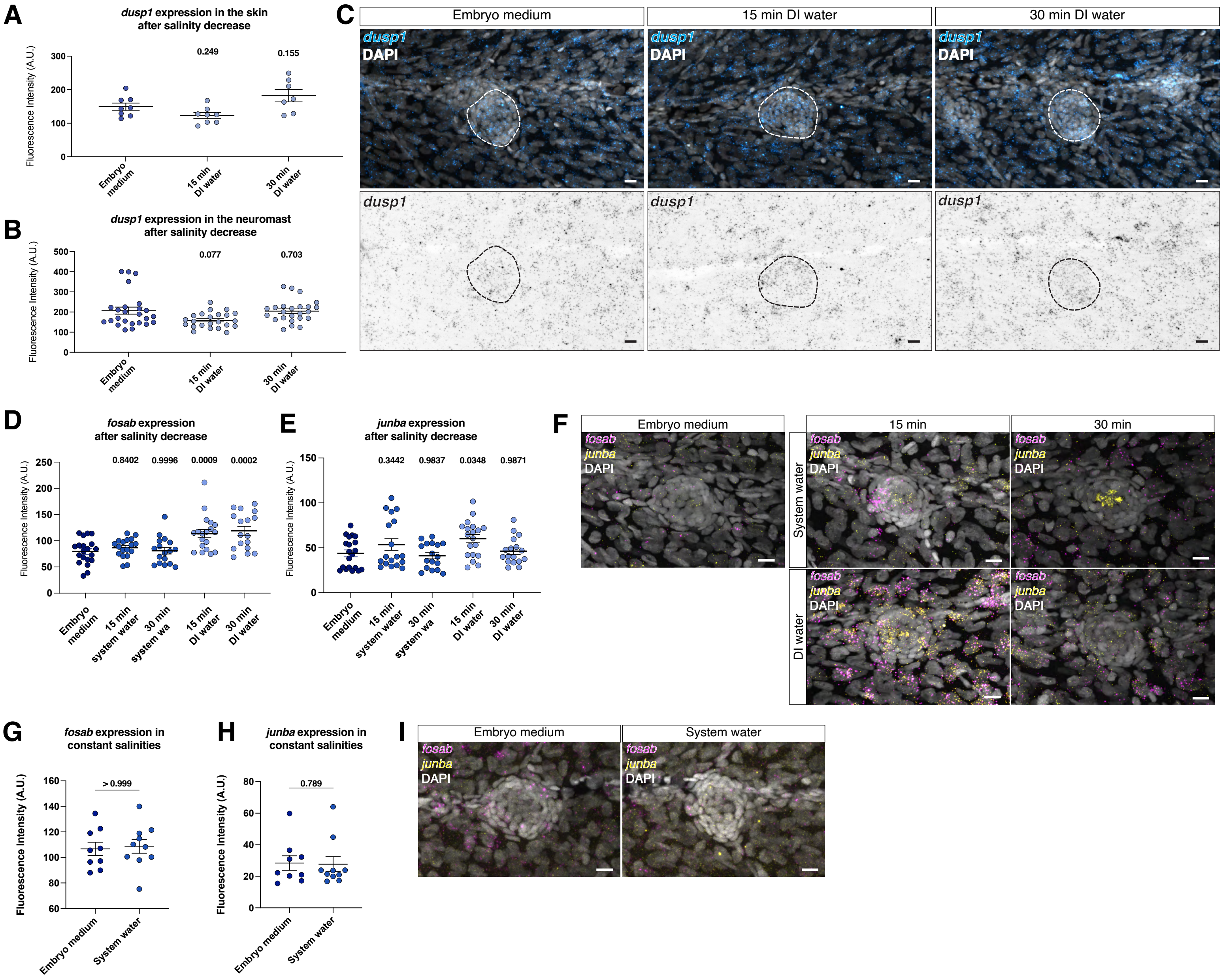

Fig. S6. Nm ionocytes are not induced by stress response pathways

(A) Expression of the GR target gene *dusp1* in the skin and (B) neuromasts quantified by HCR at different times after low salinity incubation (embryo medium n = 4 larvae, 25 neuromasts, 15 min n = 5 larvae, 23 neuromasts and 30 min n = 5 larvae, 24 neuromasts, Kruskall-Wallis ANOVA) (C) Representative images from A and B. (D) Expression of *fosab* and (E) *junba* after 15 and 30 min of salinity decrease (Kruskall-Wallis ANOVA with Dunn's multiple comparisons test). (F) Representative images from (C) and (D). (G) Expression of *fosab* and (H) *junba* in embryos raised in different salinities from the one cell stage (Mann-Whitney and Unpaired t-test, respectively). (I) Representative images from (G) and (H). Scale bars = 10 μm.

Development • Supplementary information

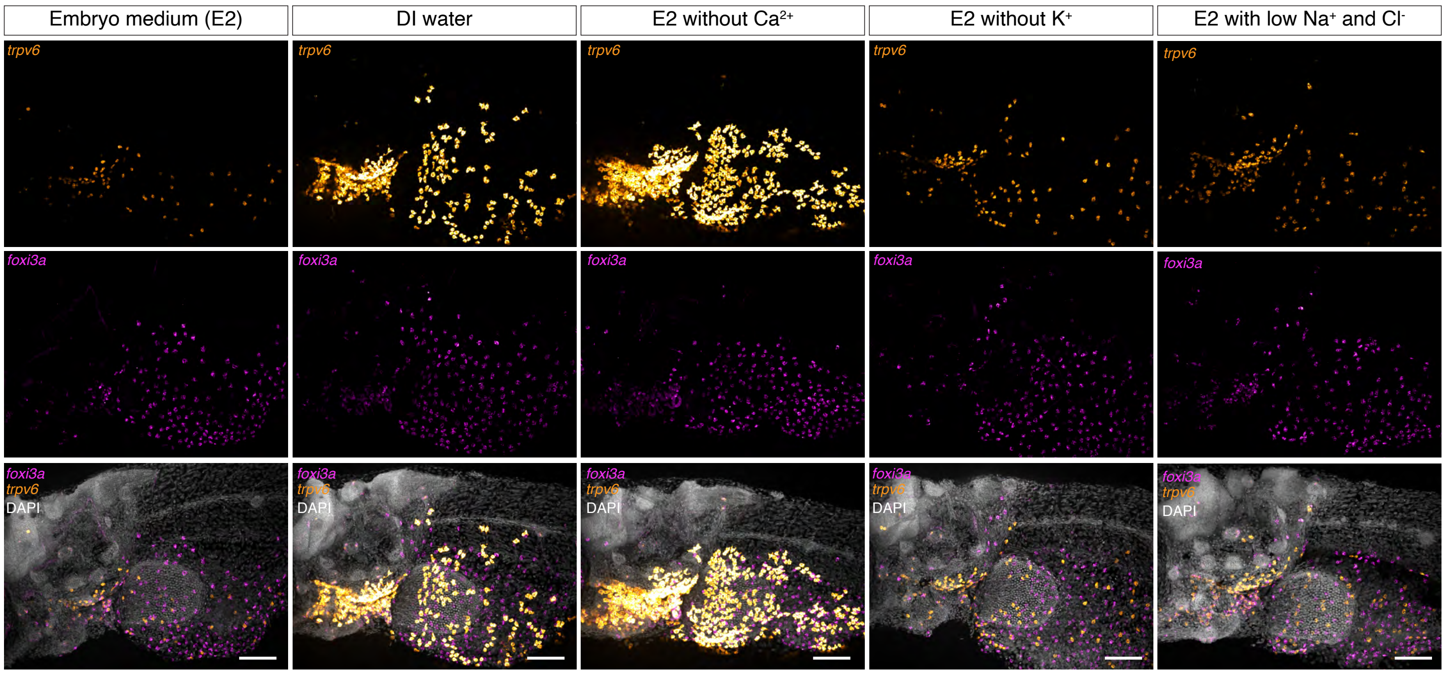

Fig. S7. Skin ionocytes also respond to specific ion depletions

foxi3a (HR ionocytes) and *trpv6* (NaR ionocytes) HCRs showing response of HR and NaR ionocytes, to 48h incubation in embryo medium, DI water, depletion of Ca^{2+}, K^{+} and low Na^{+}/Cl^{-}, scale bars = 100 μm.

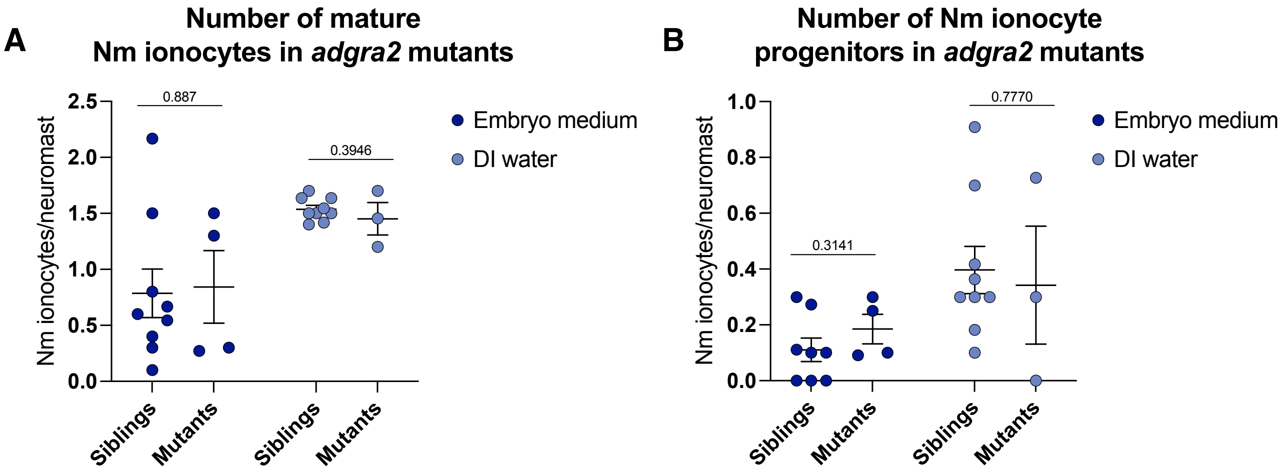

Fig. S8. Nm ionocyte recruitment does not require sensory neurons

(A) Number of mature Nm ionocytes (B) and progenitors in *adgra2* mutants (siblings in embryo medium, n = 9 larvae, 86 neuromasts, mutants in embryo medium, n = 4 larvae, 43 neuromasts, siblings in DI water, n = 9 larvae, 94 neuromasts, mutants in DI water, n = 3 larvae, 31 neuromasts, unpaired t-test).

Development • Supplementary information

Figure S9

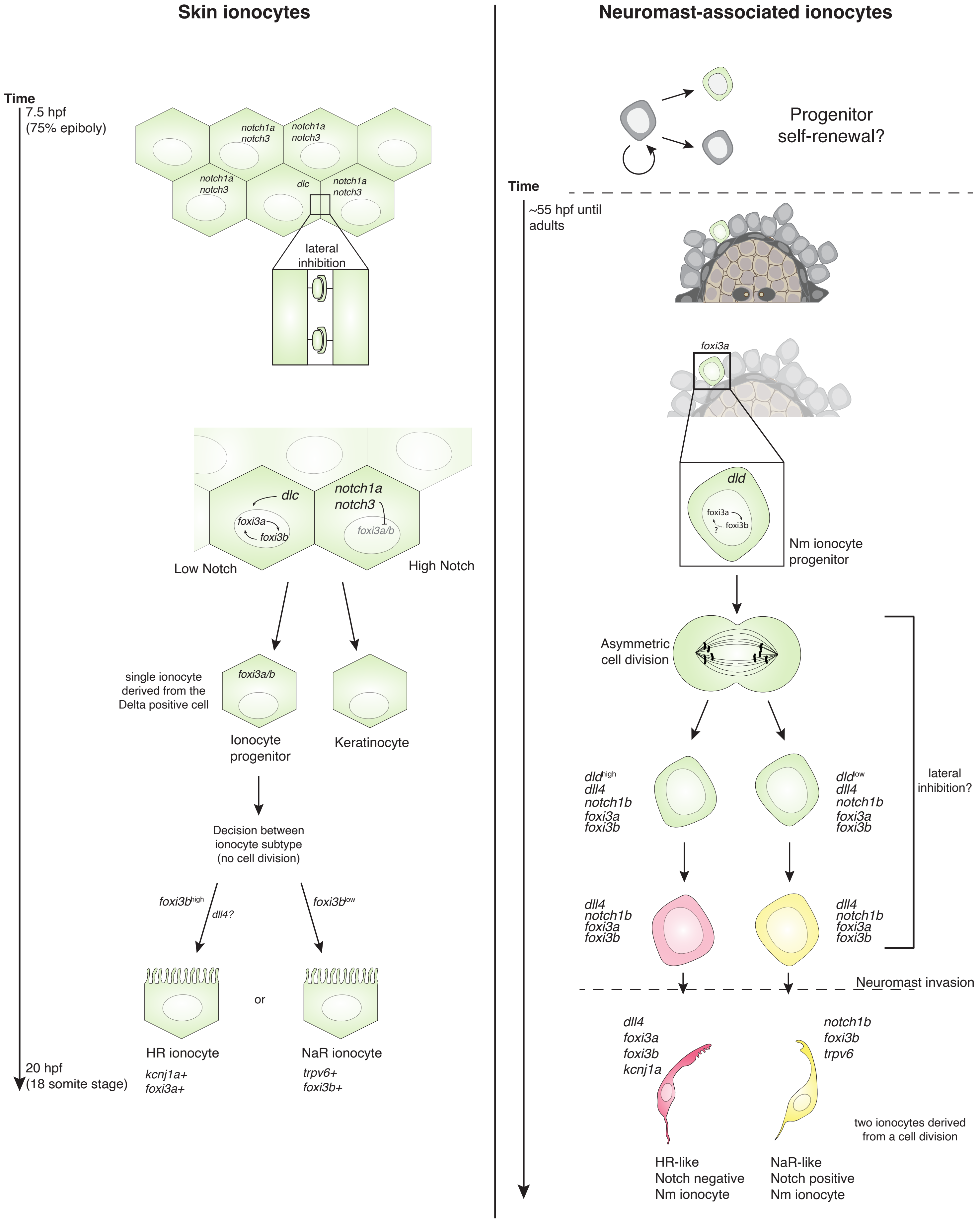

Development • Supplementary information

Fig. S9. Comparison of ionocyte differentiation in the skin and the neuromasts

Model of tissue-specific ionocyte differentiation in zebrafish. Skin ionocytes are derived from *dlc* positive cells, and one ionocyte progenitor is formed without a cell division. Skin ionocyte progenitors then become either a NaR or HR ionocyte based on the expression of *foxi3b* and/or *dll4* (based on results from Esaki et al., 2009; Hsiao et al., 2007; Jänicke et al., 2007). Nm ionocytes, on the other hand, are derived from a cell division and give rise to a Nm ionocyte pair. The resulting two cells consist of one Notch-positive (*notch1b*) and one Notch-negative (*dll4*) cell, and sustained Notch signaling controls their survival. It is known that progenitors are derived from *krtt1c19e*-positive skin cells, but how the self-renewal of this single-cell progenitor takes place is not understood.

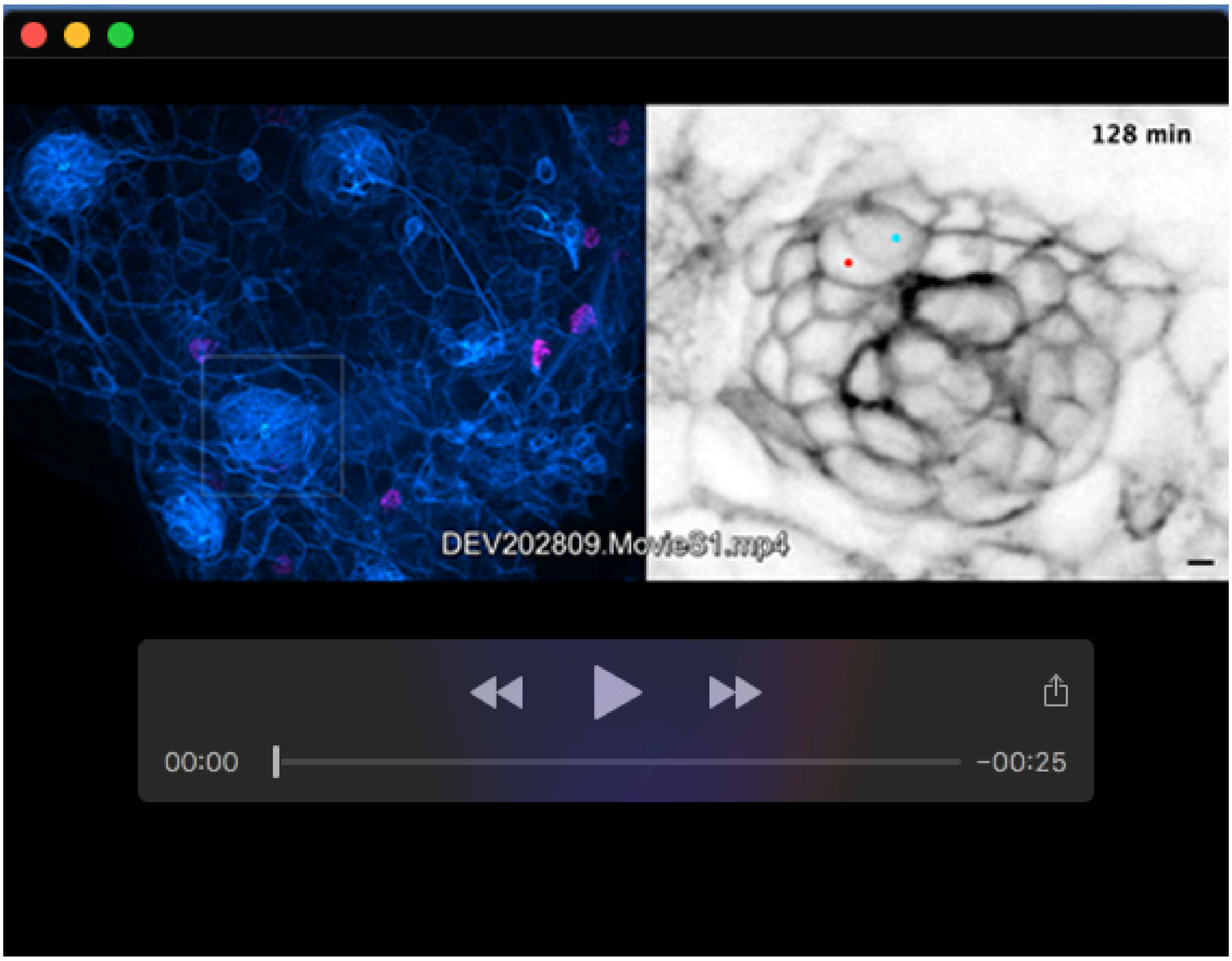

Movie 1. Skin ionocytes do not invade lateral line neuromasts. Skin ionocytes labeled by a MitoTracker pulse are not highly migratory and do not invade lateral line neuromasts at 3 dpf. Scale bar = 10 μm.

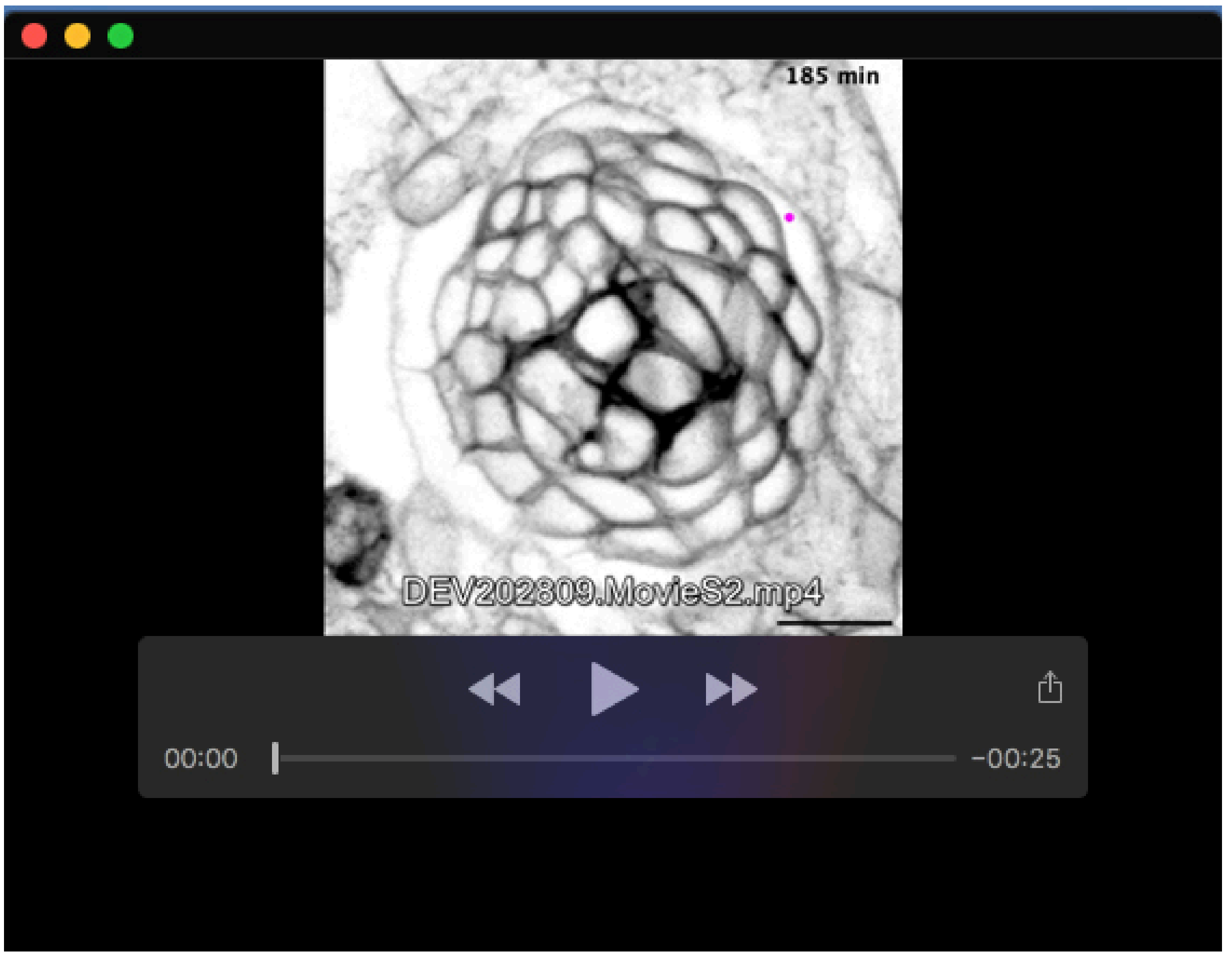

Movie 2. Nm ionocytes are derived from a cell division. Neuromast in a 3 dpf *Tg(-8.0cldnB:lyn-EGFP)*^{zf106Tg} zebrafish larva. A Nm ionocyte progenitor is seen dividing and the resulting pair invades the neuromast. Scale bar = 10 μm.

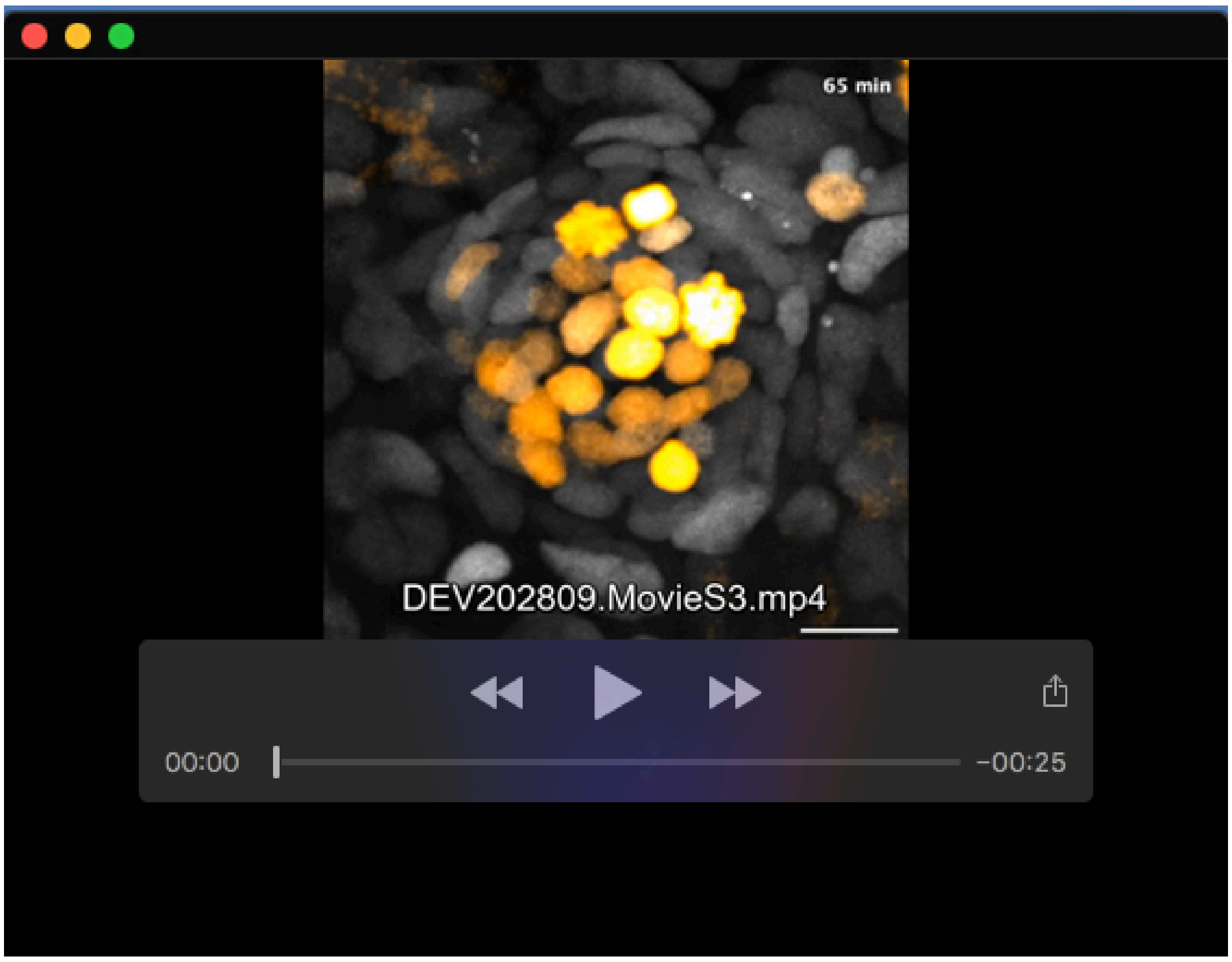

Movie 3. *dld* is upregulated prior to progenitor cell division. Time-lapse of a *dld:H2B-EGFP;ubi:H2A-mCherry* 3 dpf transgenic zebrafish shows *dld* is upregulated during cytokinesis. Scale bar = 10 μm. Gamma was changed in the *dld:H2B-EGFP* channel to allow for visualization of dim structures.

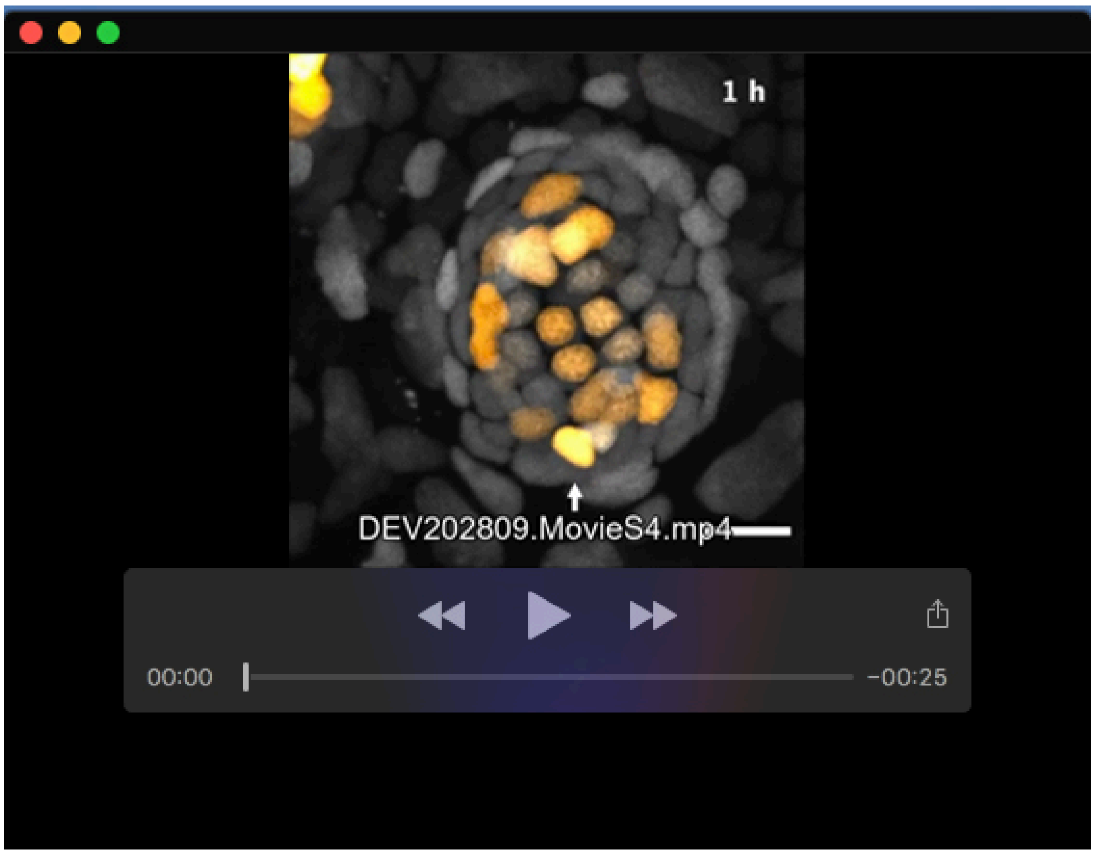

Movie 4. Nm ionocytes in a 4 dpf *dll4* sibling. Time-lapse of a *dld:H2B-EGFP;cldnb:H2A-mCherry* transgenic larva shows a mature Nm ionocyte pair over the course of 32 hours. Scale bar = 10 μm. Gamma was changed in the *dld:H2B-EGFP* channel to allow for visualization of dim structures.

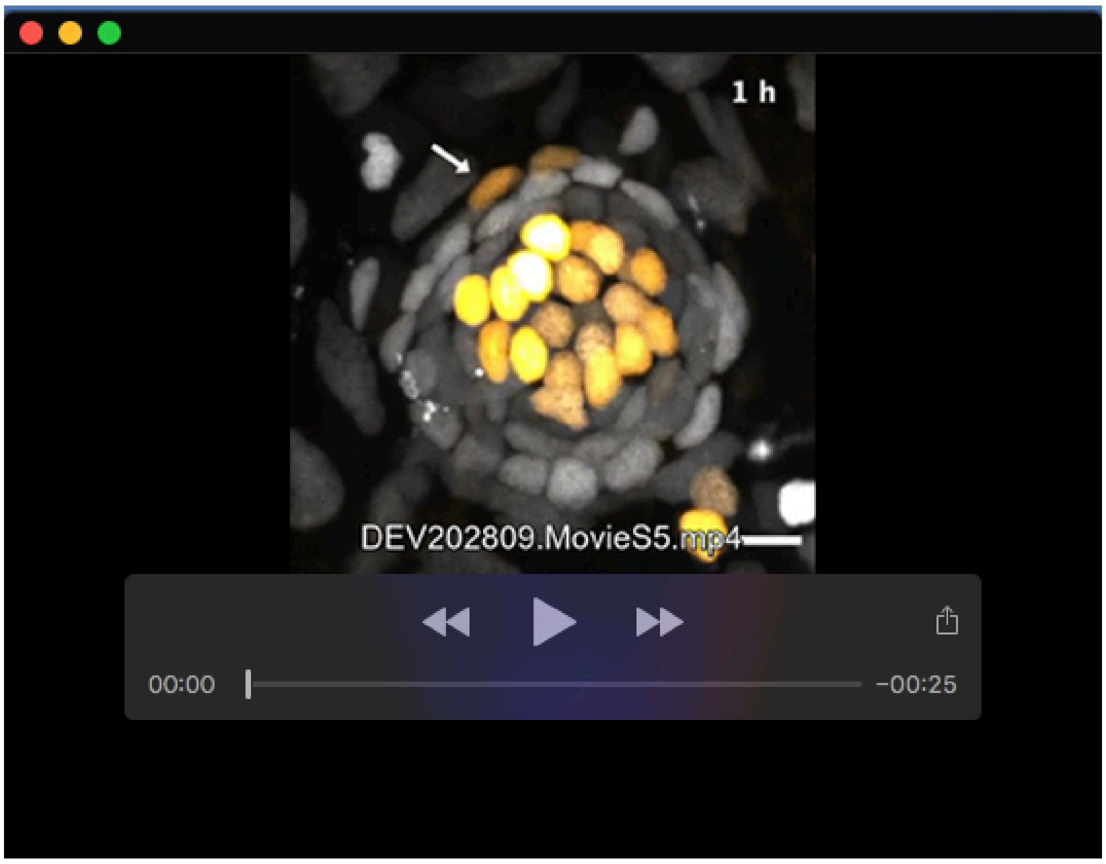

Movie 5. Cell death of Nm ionocytes is observed in 4 dpf *dll4* mutants. Time-lapse of a *dll4* mutant larva showing a pair of Nm ionocytes, labeled by *dld:H2B-EGFP*, that migrate into a neuromast and die shortly after differentiation. Scale bar = 10 μm. Gamma was changed in the *dld:H2B-EGFP* channel to allow for visualization of dim structures.

Development • Supplementary information
