# Supplementary material for: Environmental and molecular control of tissue-specific ionocyte differentiation in zebrafish
Source: Development. 2024 Oct 22;151(20):dev202809. doi: 10.1242/dev.202809 (PMC11528218; doi:10.1242/dev.202809)
Supplement: Supplementary file 1 [file develop-151-202809_review_history.pdf]

Environmental and molecular control of tissue-specific ionocyte differentiation in zebrafish

Julia Peloggia, Mark E Lush, Ya-Yin Tsai, Christopher Wood and Tatjana Piotrowski
DOI: 10.1242/dev.202809

Editor: Steve Wilson

Review timeline
Original submission: 21 February 2024
Editorial decision: 13 May 2024
First revision received: 12 August 2024
Accepted: 17 September 2024

Original submission

First decision letter

MS ID#: DEVELOP/2024/202809

MS TITLE: Environmental and molecular control of tissue-specific ionocyte differentiation in zebrafish

AUTHORS: Julia Peloggia, Mark E Lush, Ya-Yin Tsai, Christopher Wood, and Tatjana Piotrowski

Many apologies again for the extremely long time that it took us to obtain reviews on your manuscript. However, I have now received all the referees' reports, and have reached a decision. The referees' comments are appended below, or you can access them online: please go to BenchPress and click on the 'Manuscripts with Decisions' queue in the Author Area.

As you will see, the referees express considerable interest in your work, but have some criticisms and suggestions for improving your manuscript. If you are able to revise the manuscript along the lines suggested, I will be happy receive a revised version of the manuscript. Please note that Development will normally permit only one round of major revision. If it would be helpful, you are welcome to contact us to discuss your revision in greater detail. Please send us a point-by-point response indicating your plans for addressing the referees' comments, and we will look over this and provide further guidance.

Please attend to all of the reviewers' comments and ensure that you clearly highlight all changes made in the revised manuscript. Please avoid using 'Tracked changes' in Word files as these are lost in PDF conversion. I should be grateful if you would also provide a point-by-point response detailing how you have dealt with the points raised by the reviewers in the 'Response to Reviewers' box. If you do not agree with any of their criticisms or suggestions please explain clearly why this is so.

Reviewer 1

Advance summary and potential significance to field

Peloggia and colleagues expand on their previously published studies on the migration of ionocytes from skin into the neuromasts of the zebrafish lateral line system. Here they describe ionocyte precursors on the periphery of the neuromast that divide to give rise to migrating ionocytes. They describe expression of Notch ligands, receptors and downstream transcription factors, finding that

© 2024. Published by The Company of Biologists under the terms of the Creative Commons Attribution License (https://creativecommons.org/licenses/by/4.0/).

the ligands dld and dll4 are expressed in precursors. Mutation of dll4 but not dld results in loss of neuromast ionocytes. In contrast loss of dll4 alters skin ionocyte subtype, highlighting differences between the different ionocyte populations. Ionocyte precursors rapidly increase after changes in salinity. Neuromast ionocyte numbers are also altered by mutations affecting lateral line hair cell activity. Together these studies further our understanding of how environmental changes result in physiological adaptation in this sensory system.

Comments for the author

1. The evidence that osmotic cues are not involved in ionocyte number is incomplete. I am a little confused by the design of the experiments replacing ions with sucrose or sorbitol (Fig 5b). As far as I can tell there is no evidence for reducing ionocyte numbers by increasing salinity above that found in embryo medium, rather that lowering salinity induces ionocytes. A better experiment would be to develop a medium that is comparable to embryo medium osmolarity using sorbitol, that is, remove embryo medium ions but retain equivalent osmolarity.

2. Removal of calcium from the medium is often used to inactivate hair cells by disrupting the cadherin based tip links. The current experiments do not distinguish whether the effects of calcium removal are on hair cells or independent. These could be assessed by exposure to different concentrations of calcium ions.

3. Is there a positive control to demonstrate functionality of the dld overexpression construct?

4. The molecular nature of the CRISPR lesions should be reported for mutants generated in this study.

5. Figures 2, 3 – how old were the fish in these experiments?

6. Figure 3 F – Why is there no difference in the number of ionocyte progenitors comparing embryo medium with water (for either siblings or dll4 mutants)?

Reviewer 2

Advance summary and potential significance to field

In a previous study (Dev. Cell, 2021), this group has reported that skin-derived motile cells migrate/invade into lateral line neuromasts as an adaptive repones to low pH and reduced ion concentrations. In this follow-up study, the authors investigated the signals that trigger the adaptive proliferation and differentiation of these so called Nm ionocytes. They suggested that the Nm ionocytes are derived from progenitor cells located outside of the neuromasts and they undergo proliferate and migration in response to low Ca^{2+} and low pH. They explored the role of Notch signaling in regulating Nm ionocyte differentiation and survival. Finally, they presented some data that Nm ionocyte number increased in the pcdh15a mutant and concluded that sensory cell activity regulates Nm ionocyte number. Overall, this manuscript contains new and original data and addresses an important question in adaptive physiology. As it stands, however, there are a number of issues.

Comments for the author

1) This manuscript is very dense and not easy to read. It covers three distinct parts and each can be further developed.

2) Important technical data are not provided. For instance, a central part of the paper is about Notch signaling. Yet, there is no data on the characterization and validation of the did and dll4 mutant lines nor the new transgenic/knock-in lines.
Without these validation data, it is difficult to agree or disagree with the conclusions about Did or Dll4 function.

© 2024. Published by The Company of Biologists under the terms of the Creative Commons Attribution License (https://creativecommons.org/licenses/by/4.0/).

3) The conclusion that sensory cell activity regulates Nm ionocyte number is based on a single line of evidence - Nm ionocyte number increased in the pcdh15a mutant.
More independent evidence is needed.

4) Based on the data presented, it seems the notch(-) mature ionocyte is NaR ionocyte and notch(+) ionocytes may be HR cells. If this is correct, please just call them so. I understand there are minor differences in gene expression from earlier studies. But the present study used a more sensitive technique, while earlier reports used conventional whole mount in situ assays.

5) The original of Nm ionocytes and the relationship between these Nm ionocytes and skin ionocytes are confusing. Ionocytes were derived from epithelial stem cells early in development. The adaptive changes in the so-called Nm ionocytes were studied at a later stage. In their Dev. Cell, 2021 paper, it has reported that skin-derived motile cells migrate/invade into lateral line neuromasts and become Nm ionocytes. I find that model shown in Fig. S8 problematic. Are there other possibilities? Given the data shown in Fig. S6, is it possible a similar process occurred in/around neuromasts or alternatively the highly dividing NaR cells migrating into the neuromasts?

Reviewer 3

Advance summary and potential significance to field

In their manuscript, Peloggia et al. build upon their 2001 study, which first introduced the concept of adaptive cell invasion as a mechanism for maintaining lateral line organ homeostasis in response to changes in salinity. In this current work, the authors delve deeper into the environmental cues that trigger this adaptive response and elucidate the signaling mechanisms that underlie it.

The manuscript is nicely written, providing a clear rationale for the experimental design. Also noteworthy are the high-quality images showing gene expression in neuromasts, the tool development for gain and loss of function experiments, and the rigorous quantification of data.

However, several key points merit further discussion and clarification. Firstly, the interpretation of the high-resolution HCR data relies on the ability to differentiate individual cell outlines. Given the absence of a cell surface marker, the authors should elaborate on the methodology used to interpret this data accurately.

Secondly, with regard to the unidentified signal modulating the recruitment of ionocytes into neuromasts under low salinity conditions, it would be beneficial for the authors to explore the possibility of a cell-autonomous signal originating within the ionocytes themselves.

Furthermore, while the focus of the study is understandably on the adaptive responses to low salt environments, it is worth considering the broader implications for zebrafish, which are euryhaline and can adapt to a wide range of salinity levels. A discussion on whether neuromast ionocytes contribute to adaptation across various salinity conditions could enrich the manuscript.

Finally, the authors could expand on the implications of their findings by speculating on the presence of ionocytes in the zebrafish inner ear and their potential association with hair cells. Additionally, addressing whether adaptive cell invasion is specific to ionic homeostasis in open systems such as the zebrafish lateral line, or if it may have broader implications for other physiological systems, would further enhance the impact of this study.

Comments for the author

Suggestions for improving the manuscript, outlined in the section above, are discussion based and do not involve any additional experiments.

First revision

© 2024. Published by The Company of Biologists under the terms of the Creative Commons Attribution License (https://creativecommons.org/licenses/by/4.0/).

Author response to reviewers' comments

Point-by-point response to the reviewers

We appreciate the time and effort the reviewers took in crafting their thoughtful comments and for their overall very positive evaluation of our manuscript. We have taken the reviewers' comments and suggestions to heart and performed additional experiments, added figures and clarified or rephrased the text.

****** **Reviewer 1**

1. The evidence that osmotic cues are not involved in ionocyte number is incomplete. I am a little confused by the design of the experiments replacing ions with sucrose or sorbitol (Fig 5b). As far as I can tell there is no evidence for reducing ionocyte numbers by increasing salinity above that found in embryo medium, rather that lowering salinity induces ionocytes. A better experiment would be to develop a medium that is comparable to embryo medium osmolarity using sorbitol, that is, remove embryo medium ions but retain equivalent osmolarity.

- We thank the reviewer for their comments. We agree that the evidence that osmolarity does not affect Nm ionocyte numbers should be strengthened. To address the reviewer's comment, we added the suggested experiment in which we supplemented DI water with sorbitol and sucrose to the same osmolarity as E2 medium (Figure 5C). We observed higher numbers of Nm ionocyte in these solutions when compared to embryo medium. The results confirm that Nm ionocyte number is osmolarity-independent and nicely supports the experiment the reviewer is referring to, in which we use isotonic solutions to determine if removing the osmolarity differences between fish and the medium would affect ionocytes. Together, these experiments strongly suggest that ion concentration, rather than osmolarity, regulates Nm ionocyte number.

2. Removal of calcium from the medium is often used to inactivate hair cells by disrupting the cadherin based tip links. The current experiments do not distinguish whether the effects of calcium removal are on hair cells or independent. These could be assessed by exposure to different concentrations of calcium ions.

- We agree with the reviewer that our ion depletion treatments may be regulating Nm ionocyte number via affecting hair cell activity. The reviewer suggested to perform different concentration of calcium ions, however, this experiment has the caveat that Nm ionocyte number itself is concentration dependent (Peloggia and Münch et al., 2021). We instead decided to treat *pcdh15a* mutants with calcium-depleted medium. Hair cells are not functional in these mutants, so any effect on ionocyte number after calcium depletion would be caused by a hair-cell independent effect.

Our results, summarized in Figure 5E and lines 327-332, show that *pcdh15a* mutants treated with calcium-depleted medium respond similarly to those treated with DI water, suggesting that calcium depletion, while likely also affecting hair cell function, regulates Nm ionocyte number through a mechanism independent of hair cells.

3. Is there a positive control to demonstrate functionality of the *dld* overexpression construct?

Functionality of the *dld* overexpression construct is supported by the upregulation of *dld* mRNA in the old Supplemental Figure 5. However, although we believe that our transgenic line is working, we chose to remove the data from the manuscript, as the result is difficult to interpret. Overexpression of *dld* in all cells may lead to inhibition of Notch signaling through cis-inhibition, and not an activation like we initially intended.

4. The molecular nature of the CRISPR lesions should be reported for mutants generated in this study.

© 2024. Published by The Company of Biologists under the terms of the Creative Commons Attribution License (https://creativecommons.org/licenses/by/4.0/).

- We thank the reviewer for catching this omission and we added more detailed information regarding the nature of the mutations. We have now included thorough analyses of the *dld* and *dll4* mutants, showing additional phenotypes and HCRs of the mutated genes. Both mutations lead to a complete loss of the transcripts, now shown in Supplemental Figures 4 and 5.

5. Figures 2, 3 – how old were the fish in these experiments?

- We have added to the figure legends that fish in these experiments are all 4-5 dpf.

6. Figure 3 F – Why is there no difference in the number of ionocyte progenitors comparing embryo medium with water (for either siblings or dll4 mutants)?

- Thank you for pointing this out. In our previous analyses of this mutant, we did not take the high variability of the data into account. Upon closer inspection of our data, we observed that in some cases, such as Figure 3F, variability between the same condition in different replicates was very high, obfuscating the differences between embryo medium and DI water. In all replicates, however, DI water incubation did lead to an increase in Nm ionocyte progenitors. We also performed an additional replicate, which has been added to the figure, and saw the same trend of an increase after DI water. To compensate for such variability, we now employed a normalization method in which we divide all values by the average of control group. For experiments in which the variability was not as high, we opted to leave the raw data, as the numbers are more informative to the reader. Normalized data points (also Figures 5A and 5E) have been clearly stated in their figure legends and in the Material and Methods (lines 638-641).

****** **Reviewer 2**
Overall, this manuscript contains new and original data and addresses an important question in adaptive physiology. As it stands, however, there are a number of issues.

1) This manuscript is very dense and not easy to read. It covers three distinct parts and each can be further developed.

- We thank the reviewer for their feedback. We have edited and hopefully improved the readability of the manuscript.

2) Important technical data are not provided. For instance, a central part of the paper is about Notch signaling. Yet, there is no data on the characterization and validation of the dld and dll4 mutant lines nor the new transgenic/knock-in lines. Without these validation data, it is difficult to agree or disagree with the conclusions about Dld or Dll4 function.

- We thank the reviewer for their comment, which was also noted by reviewer 1. We now include a much more detailed characterization of the mutants and transgenics. For the CRISPR mutants, we now show in Supplemental Figures 4 and 5 images of additional phenotypes and HCRs that demonstrate that the mutations lead to a loss of transcripts. For the *dld*:H2B-EGFP, we show the co-localization of the endogenous *dld* transcript with *egfp* mRNA, as well as the overlap of *dld* mRNA expression and EGFP protein in whole embryos. For the *hsp70:dld* transgenic line, we opted to remove the data from the manuscript as the results are more difficult to interpret than we initially anticipated.

3) The conclusion that sensory cell activity regulates Nm ionocyte number is based on a single line of evidence - Nm ionocyte number increased in the pcdh15a mutant. More independent evidence is needed.

As an independent way of testing the role of hair cell function in Nm ionocyte development, we disrupted hair cell function using a pharmacological approach by soaking the larvae in the drug benzamil, which is commonly used in the field as a potent hair cell inhibitor (Hailey et al., 2017;

© 2024. Published by The Company of Biologists under the terms of the Creative Commons Attribution License (https://creativecommons.org/licenses/by/4.0/).

Pickett et al., 2019). Treatment of larvae with benzamil indeed decreases DASPEI uptake in hair cells and increases Nm ionocyte number (see image below). However, a caveat of pharmacological inhibition of hair cell function is that these treatments affect ion uptake. Specifically, benzamil affects primarily sodium uptake, and could likely do so in both the hair cells and ionocytes. Therefore, pharmacological inhibitors are not adequate tools to specifically manipulate hair cell function, and we did not include these data in the manuscript.

NOTE: We have removed unpublished data that had been provided for the referees in confidence.

A possible caveat of the *pcdh15a* mutants could be if *pcdh15a* was also expressed in other cell types that could influence ionocyte numbers, or if it was expressed in ionocytes themselves. We therefore analyzed different scRNA-seq datasets and observed that *pcdh15a* is not expressed in any ionocyte or skin cell types. This further suggests that the increase in ionocyte numbers in *pcdh15a* mutants is due to specific loss of hair cell function.

Below is the UMAP containing different ionocytes, skin cell and neuromast cell types (left) and a Feature Plot showing *pcdh15a* expression (right). The red circle highlights the hair cell cluster, which is the only cluster expressing *pcdh15a*.

NOTE: Figure provided for reviewer has been removed. It showed Figure 1N from Peloggia J.*, Münch D.*, Meneses-Giles P., Romero-Carvajal A., McClain M., Pan, Y.A., Piotrowski T. (2021). Adaptive cell invasion maintains lateral line organ homeostasis in response to environmental changes. Dev Cell. 2021 May 3;56(9):1296-1312.e7. doi: 10.1016/j.devcel.2021.03.027. PMID: 33878346. We have removed unpublished data that had been provided for the referees in confidence.

As *pcdh15a* is exclusively expressed in hair cells and affects hair cell function (reviewed in Sakaguchi et al., 2009), we believe that it is an excellent tool to show that hair cell function is involved in increasing Nm ionocyte numbers, even though other cells must also be involved. Additionally, we now discuss that this effect may not be direct (lines 380-391).

4) Based on the data presented, it seems the notch(-) mature ionocyte is NaR ionocyte and notch(+) ionocytes may be HR cells. If this is correct, please just call them so. I understand there are minor differences in gene expression from earlier studies. But the present study used a more sensitive technique, while earlier reports used conventional whole mount in situ assays.

- We thank the reviewer for their feedback and changed in the text the Nm ionocyte naming to HR- and NaR-like Nm ionocytes.

5) The origin of Nm ionocytes and the relationship between these Nm ionocytes and skin ionocytes are confusing. Ionocytes were derived from epithelial stem cells early in development. The adaptive changes in the so-called Nm ionocytes were studied at a later stage. In their Dev. Cell, 2021 paper, it has reported that skin-derived motile cells migrate/invade into lateral line neuromasts and become Nm ionocytes. I find that model shown in Fig. S8 problematic. Are there other possibilities? Given the data shown in Fig. S6, is it possible a similar process occurred in/around neuromasts or alternatively the highly dividing NaR cells migrating into the neuromasts?

We previously showed that Nm ionocytes are not derived from skin ionocytes. We agree, however, that we currently lack lineage tracing tools to determine if skin and Nm ionocytes share a common progenitor, or if there is a progenitor population surrounding the neuromast that is specific to Nm ionocytes. As skin and Nm ionocytes have many genes in common, we nevertheless believe that it is worth to build hypothetical gene regulatory networks and compare them between the two different ionocyte subtypes, particularly because our data suggests that some of the upstream factors, such as the specific Notch pathway members, are different between the two types. We have rephrased the text to reflect that these are hypothetical models and that different possible explanations might exist.

© 2024. Published by The Company of Biologists under the terms of the Creative Commons Attribution License (https://creativecommons.org/licenses/by/4.0/).

Below we summarize our data that supports that Nm ionocytes are not derived from earlier developing skin ionocytes:

- Skin ionocytes derive from *tp63*$^{+}$/*krtt1c19e*$^{+}$ cells early in development, as detected by *foxi3a in situ hybridization* as early as 90% epiboly (Hsiao et al., 2007). In contrast, the earliest we observe Nm ionocytes is around 60hpf. We have previously shown (Peloggia and Münch et al., 2021) that, by using Cre-based lineage tracing in combination with a *krtt1c19e* promoter, which only becomes active around 24hpf, skin ionocytes are not labeled, but Nm ionocytes are (Lee et al., 2014). This was the first evidence that Nm ionocytes are likely not derived from differentiated skin ionocytes.

Another piece of evidence that Nm ionocytes are not derived from skin ionocytes is based on differences in cell size, morphology and expression between Nm ionocyte progenitors and skin ionocytes (Figures 1E-G and S1A-H).

It has been shown that skin NaR ionocytes re-enter the cell cycle in low calcium conditions (Xin et al., 2019). In contrast, we have not observed any cell divisions of mature Nm ionocytes in any of the conditions analyzed so far (larvae, homeostasis and low salinity), but rather new invasion events by a new progenitor pair.

To confirm our conclusion that skin ionocytes do not invade neuromasts we took time lapses of labeled mature skin ionocytes. At 32hpf, when skin ionocytes are mature, we stained larvae with a pulse of MitoTracker, which labels skin ionocytes (Esaki et al., 2009; Shir-Mohammadi and Perry, 2020). We then incubated the stained fish in DI water and observed that only non-labeled skin cells invaded neuromasts (Supplemental Figure 1I and 1J). Furthermore, MitoTracker-labeled skin ionocytes are relatively stationary cells that did not migrate during our time lapses.

We opted to leave the model Supplemental Figure 9 in the manuscript as a comparison of the hypothetical GRNs. We thought it would be useful for the field to have models for each ionocyte cell type that show the differences and commonalities that can now be tested experimentally.

****** **Reviewer 3**

The manuscript is nicely written, providing a clear rationale for the experimental design. Also noteworthy are the high-quality images showing gene expression in neuromasts, the tool development for gain and loss of function experiments, and the rigorous quantification of data. However, several key points merit further discussion and clarification.

Firstly, the interpretation of the high-resolution HCR data relies on the ability to differentiate individual cell outlines. Given the absence of a cell surface marker, the authors should elaborate on the methodology used to interpret this data accurately.

- We thank the reviewer for the positive comments on our manuscript. We have now included in our methods section a description of how we performed the HCR quantification (lines 683-691).

To determine expression of HCR probes at the single cell level in mature ionocytes, we used the Notch reporter *Tg(tp1bglobin:EGFP)*^{um14}, which is expressed only in the NaR-like Nm ionocyte. For progenitors, we observed that *foxi3b* is expressed in the progenitor cell before cell division, in the two daughter cells and subsequently in both mature Nm ionocytes. Therefore, for all analyses of progenitor cells, we incorporated *foxi3b* to identify progenitor cells prior and after division.

Most of the HCRs worked really well and expression within individual cells can be easily detected (eg Figure 1D-F). For the experiments in Figures 2B, 2D and 2E, where expression of candidate genes is evaluated within the pair, we only analyzed expression that overlapped with the nuclei of individual cells.

The model Figure S9 shows genes that are specific for each ionocyte type (data in Figures 1 and 2). We were therefore able to determine the identity of each ionocyte within the pair without much difficulty.

© 2024. Published by The Company of Biologists under the terms of the Creative Commons Attribution License (https://creativecommons.org/licenses/by/4.0/).

Secondly, with regard to the unidentified signal modulating the recruitment of ionocytes into neuromasts under low salinity conditions, it would be beneficial for the authors to explore the possibility of a cell-autonomous signal originating within the ionocytes themselves.

We agree that there is a high chance for the environmental sensing to be ionocyte cell-autonomous. We are now discussing this possibility in lines 380-391.

Furthermore, while the focus of the study is understandably on the adaptive responses to low salt environments, it is worth considering the broader implications for zebrafish, which are euryhaline and can adapt to a wide range of salinity levels. A discussion on whether neuromast ionocytes contribute to adaptation across various salinity conditions could enrich the manuscript.

- We fully agree with the reviewer. We have now added a paragraph that discusses Nm ionocytes, adaptability and evolution (lines 489-505).

Finally, the authors could expand on the implications of their findings by speculating on the presence of ionocytes in the zebrafish inner ear and their potential association with hair cells. Additionally, addressing whether adaptive cell invasion is specific to ionic homeostasis in open systems such as the zebrafish lateral line, or if it may have broader implications for other physiological systems, would further enhance the impact of this study.

- We agree and now have added a discussion about the role of ionocytes and ion channels in other systems, especially about the role of Pendrin (SLC26) in the ear and CFTR in the lungs (lines 458-487).

Reviewer 3 Comments for the Author:
Suggestions for improving the manuscript, outlined in the section above, are discussion based and do not involve any additional experiments.

<u>Second decision letter</u>

MS ID#: DEVELOP/2024/202809

MS TITLE: Environmental and molecular control of tissue-specific ionocyte differentiation in zebrafish

AUTHORS: Julia Peloggia, Mark E Lush, Ya-Yin Tsai, Christopher Wood, and Tatjana Piotrowski
ARTICLE TYPE: Research Article

I am happy to tell you that the referees are happy with your revisions and your manuscript has been accepted for publication in Development, pending our standard publication integrity checks.

<u>Reviewer 1</u>

Advance summary and potential significance to field

The authors have strengthening an already well-constructed study.

Comments for the author

They have addressed my previous concerns.

<u>Reviewer 2</u>

© 2024. Published by The Company of Biologists under the terms of the Creative Commons Attribution License (https://creativecommons.org/licenses/by/4.0/).

Advance summary and potential significance to field

See my previous review comments

Comments for the author

The authors did a nice job in revising this MS. I have no more major concerns.

Reviewer 3

Advance summary and potential significance to field

The authors have thoroughly addressed my previous concerns, as well as those raised by other reviewers, resulting in significant improvements to the manuscript. The revisions have enhanced both the clarity and quality of the work.
I believe the manuscript is now well-suited for publication and will be highly valuable to those interested in development, environmental influences on development, and sensory signaling more broadly

Comments for the author

No further comments.

© 2024. Published by The Company of Biologists under the terms of the Creative Commons Attribution License (https://creativecommons.org/licenses/by/4.0/).
